# Genomic Data Reveal Multiple Introduction Sources and Limited Post‐Colonization Gene Flow in Southeast Michigan Invasive Red Swamp Crayfish (*Procambarus clarkii*)

**DOI:** 10.1002/ece3.72550

**Published:** 2025-12-09

**Authors:** Nicole E. Adams, Jared J. Homola, Nicholas M. Sard, Lucas R. Nathan, Brian M. Roth, John D. Robinson, Kim T. Scribner

**Affiliations:** ^1^ Department of Fisheries and Wildlife Michigan State University East Lansing Michigan USA; ^2^ Biological Sciences Department The State University of New York—Oswego Oswego New York USA; ^3^ Fisheries Division Michigan Department of Natural Resources Lansing Michigan USA

**Keywords:** Approximate Bayesian Computation, bridgehead effect, demographic modeling, gene flow, invasive species, landscape genetics, *Procambarus clarkii*

## Abstract

Understanding the colonization history and factors associated with changes in distribution and abundance of invasive species is paramount to inform control measures that prevent further spread and facilitate eradication. We investigated spatial patterns in genetic diversity and differentiation of the globally invasive species, the Red Swamp Crayfish ( *Procambarus clarkii* ) at a recent invasion front in the Detroit metropolitan area of southeast (SE) Michigan, USA. Genomic data based on collections of 763 individuals from 20 waterbodies and 2675 single nucleotide polymorphism (SNP) loci were used to estimate genetic diversity and spatial genetic structure and to test the hypotheses of gene flow and secondary spread. We identified strong genetic structure, and demographic (coalescent) analysis supported models consistent with multiple introductions from separate genetic lineages. Across the study area, seven genetically distinct clusters were identified. We found evidence of limited dispersal consistent with an isolation‐by‐distance pattern of gene flow among waterbodies. We also detected evidence of secondary dispersal from early invasive populations consistent with a bridgehead effect. Local landscape features including hydrological features, major land use types, and roads were not predictive of spatial genetic relationships among waterbodies within geographic clusters. Results suggest that the *P. clarkii* invasion of SE Michigan has proceeded through a combination of repeated introductions and secondary spread at fine spatial scales. The highly developed landscape of the metropolitan Detroit area did not appear to deter the movements of *P. clarkii* , suggesting further spread is likely.

## Graphical abstract

Examining the genetic patterns of invasive Red Swamp Crayfish in southeast Michigan, we found evidence of multiple separate introductions along with limited natural dispersal between geographic–genetic groups. We detected evidence of secondary dispersal from early invasive populations consistent with a bridgehead effect and little evidence the heavily developed urban landscape impedes crayfish movement at fine scales. The crayfish invasion of SE Michigan has proceeded through a combination of repeated introductions and secondary spread suggesting further spread is likely.

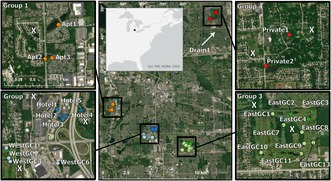

## 1 Introduction

The economic and environmental costs of invasive species are substantial and growing (Gallardo et al. 2016; Seebens et al. 2017; Seebens et al. 2018; Haubrock et al. 2021; Fantle‐Lepczyk et al. 2022). The 2005 Millennium Ecosystem Assessment recognized invasive species as one of the primary threats to global biodiversity and ecosystem function (MEA 2005). Given finite resources available for the management and control of non‐native species, appropriate responses to species invasions will often depend on the degree to which the species has become established in non‐native habitat. Management actions are best directed at detection and prevention during the early phases of an invasion, before the non‐native species has become widespread (Hobbs and Humphries 1995). Effective prevention requires accurate information on introduction routes and the potential for secondary spread from newly established populations. Accordingly, studies characterizing genetic diversity and spatial differentiation of invasive species populations are increasingly being used to improve understanding of the mechanisms associated with successful establishment and the subsequent spread in non‐native environments (e.g., Matheson and Mcgaughran 2022; Mcgaughran et al. 2024).

Genetic data can be used to characterize patterns of dispersal, demographic history, and to identify sources of invasive species, collectively informing management and eradication efforts (Sard et al. 2019; Synnott et al. 2023; Zarri et al. 2025). Dispersal ability, in particular, is identified as an important trait contributing to invasive species establishment and spread (Perkins et al. 2013). Population genetic analyses can help to quantify local population sizes, estimate rates of dispersal among populations and the speed of invasion front movement, and identify geographic origins and likely vectors of movement (e.g., Bryan et al. 2005; Brown and Stepien 2009; Estoup et al. 2010; LaRue et al. 2011; Sotka et al. 2025). Genetic information is important to improve management response by identifying promising areas to focus eradication efforts at local (i.e., natural dispersal) and long‐distance scales (i.e., human‐assisted movement). Understanding rates of dispersal and physical environmental features that facilitate or impede movements can inform managers of risks of spread and facilitate the development of effective control strategies.

**FIGURE 1:**
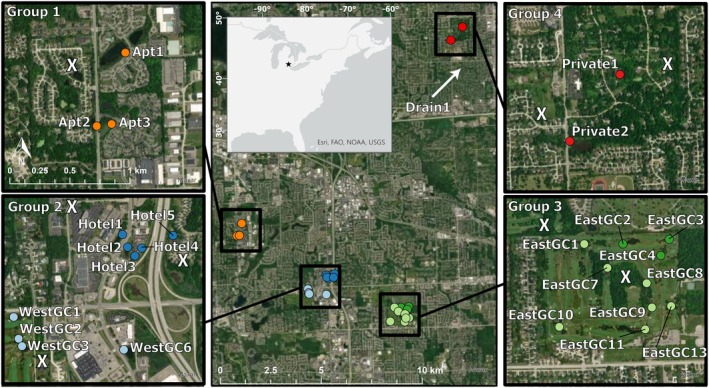
Map of waterbodies sampled for invasive *P. clarkii* in southeastern Michigan, USA. The waterbodies are shown as four geographic groups in black squares in the center map, and at an expanded geographic scale in the side panels. All four side panels share the same scale. The fifth geographic group, Drain, is 30 km northwest of Group 4, as indicated by the white arrow. White Xs indicate waterbodies that were surveyed but no *P. clarkii* were found.

The processes that underlie the introduction of non‐native species can impact the levels of genetic diversity and the likelihood of establishment. Multiple introductions from the native range, in particular, can maintain diversity in the introduced range and may be common in species invasions. Several recent studies have also provided evidence for secondary or serial invasions, in which invasive populations themselves act as sources for new introductions (Lombaert et al. 2010; Ascunce et al. 2011; Bertelsmeier and Keller 2018). Two serial invasion patterns have emerged: (1) a stepping‐stone model where dispersal and colonization facilitate spread to adjacent geographic regions and (2) a bridgehead model where an invasive population acts as a source for multiple, geographically separate populations (see Figure 1 in Lawson Handley et al. 2011). The invasive bridgehead effect model is gaining traction as an explanatory route of invasive populations (e.g., Lombaert et al. 2010; Bertelsmeier and Keller 2018; Correa et al. 2019; Blumenfeld et al. 2021; Bras et al. 2022). One study found that invasive ant species established on a greater number of continents were significantly more likely to be introduced by a bridgehead effect (Bertelsmeier and Ollier 2021). Species introduced by serial invasions may have increased introduction rates and higher survivability in part due to adaptation of already established individuals. For example, invasive cane toads in Australia have longer legs than those in the native range that may facilitate greater secondary dispersal ability (Hudson et al. 2020). In ragweed ( *Ambrosia artemisiifolia* ), rapid evolution to warmer and wetter climate in the invasive range and changes in reproductive traits during secondary spread appear to be facilitating expansion in the non‐native range (van Boheemen et al. 2019). However, strategic locations, such as major shipping ports, can lead to secondary introductions without local adaptation (Bertelsmeier and Keller 2018). Understanding the underlying invasion dynamics can lead to better, targeted management practices.

The Red Swamp Crayfish (RSC; *Procambarus clarkii* ) is a prolific and globally introduced invasive species (Gherardi et al. 2011; Loureiro et al. 2015). *P. clarkii* is native to the south‐central United States and northern Mexico but has been introduced to every continent except for Australia and Antarctica (Loureiro et al. 2015). Multiple assisted‐dispersal vectors, including the pet and live food trades, bait shops, fisheries enhancement, and live biological specimens bought from biological supply companies supplied for classroom use (Hobbs III et al. 1989; Chucholl 2013; Smith et al. 2018; Oficialdegui et al. 2020; Alvanou et al. 2024; Olden and Carvalho 2024), are contributing to the wide‐scale introductions of *P. clarkii* . Ecosystem integrity (Tricarico et al. 2010) is threatened by introduced *P. clarkii* , specifically by destabilizing native aquatic species, including macrophytes, invertebrates, amphibians, and fish (Rodríguez et al. 2003; Gherardi and Acquistapace 2007; Matsuzaki et al. 2009; Twardochleb et al. 2013; Souty‐Grosset et al. 2016). Introduced populations can be particularly damaging to native crayfish (Gil‐Sánchez and Alba‐Tercedor 2002; Holdich et al. 2009; Kendrick et al. 2024). For example, *P. clarkii* is a vector for the pathogen that causes crayfish plague ( *Aphanomyces astaci* ; Mrugała et al. 2017; Martín‐Torrijos et al. 2018).

The life history of *P. clarkii* contributes to its invasion success. *Procambarus clarkii* is a brooding freshwater crustacean that has one to two reproductive cycles per year depending on climate (Huner and Barr 1991; Adão and Marques 1993; Alcorlo et al. 2008; Adams et al. 2024). One female can produce a large number of eggs (Payne 1997; Gherardi and Acquistapace 2007; Aquiloni et al. 2005; Jin et al. 2019) often from multiple matings with different males (Yue et al. 2010; Adams et al. 2024) leading to high propagule pressure and increased within‐population genetic diversity (Sakai et al. 2001). Red swamp crayfish can survive out of water for 10 h (Anastácio et al. 2015) leading to a high capacity for passive and active dispersal. Ramalho and Anastácio et al. (2015) found *P. clarkii* to move a maximum of 90 m/h and a maximum overland distance of 21.4 m. Their movement potential and behavioral plasticity (Hanshew and Garcia 2012) are advantageous when invading new areas.

Established invasive populations of *P. clarkii* were confirmed in 2017 near Detroit, Michigan, United States (Smith et al. 2018). Current management efforts in the area involve trapping to detect newly established populations and pesticide treatments to suppress invasive *P. clarkii* populations in infested aquatic habitats (Budnick et al. 2022, 2024). Additional efforts have included burrow digging to target gravid females, carbon dioxide treatments, and pesticide treatments (unpublished data). From 2019 to 2021, there were 2197 total surveys and 63,882 traps deployed across the 25 waterbodies used in this study. Over those 3 years, a total of 123,739 *P. clarkii* were removed, with 24,670 removed from EastGC1 alone. A recent RADseq genetic study evaluated *P. clarkii* samples collected in Michigan, USA comparing them to five potential sources (Sard et al. 2023), and concluded that there were likely multiple introductions of *P. clarkii* from genetically distinct sources into the state. The study called for a more spatially comprehensive genetic investigation to evaluate fine‐scale rates and direction of dispersal, and to identify dispersal vectors (Sard et al. 2023).

In this study, we sought to use population genomic data derived from a newly developed RAD capture panel (Adams et al. 2024) to understand the genetic structure of *P. clarkii* in SE Michigan. Here we developed three research questions to quantitatively address the genetic structure of *P. clarkii* while gaining critical information regarding their invasion history in SE Michigan. First, how are populations related across the landscape? We predict based on previous work (Sard et al. 2023) that ordination and clustering methods would identify multiple unique genetic clusters within SE Michigan. Second, how does the landscape influence movement among populations? We predict that the spatial genetic structuring among infested waterbodies is statistically related to landscape‐scale features acting as barriers to gene flow, including hydrography, canopy cover, roads, and land development. Lastly, how did the colonization processes affect relationships among populations? We predict that demographic models of invasion history will show that multiple independent colonization events were followed by either a stepping‐stone or bridgehead pattern of secondary spread in SE Michigan.

## 2 Materials and Methods

### 2.1 Sampling and Sample Processing

*Procambarus clarkii* was collected from 25 waterbodies in southeast Michigan, USA, between May and October in three consecutive years (2019–2021) as part of ongoing removal efforts by the Michigan Department of Natural Resources (DNR) and Michigan State University (MSU) (Figure 1, Table S1). Most of the sampled waterbodies were non‐natural systems such as ponds located on golf courses, retention basins, and other human‐made water catchments in highly developed areas. Gee‐style minnow traps baited with dog food and unbaited artificial refuge‐style traps were checked regularly (Monday–Friday). Traps were deployed equidistantly at a density of one trap per five meters of shoreline. Thus, the total number of traps was not equal across all ponds because of differences in waterbody area. For further field method details see Budnick et al. (2024). Waterbodies were grouped into five regions, labeled Group 1 through 5, based on geographic proximity (Figure 1). Sampled locations were separated by > 2 km of terrestrial habitat, which is near the maximum extent of *P. clarkii* overland dispersal ability (Barbaresi et al. 2007).

A subset of the captured adult *P. clarkii* was preserved in 95% ethanol for genetic analysis. For each site we targeted a sample size of ≥ 30 adults. To preserve tissues for DNA extraction and genotyping, ethanol was changed within the first 5 to 7 days after collection, then again once every 2 weeks until the samples could be processed (based on previous observations of tissue degradation when left in the original ethanol). For each individual, we recorded the capture date, sex, and carapace length (mm) measured from the tip of the rostrum to the posterior end of the carapace using a digital caliper. We followed the descriptions in Huner and Barr (1991) to distinguish sexes based on gonopodia and used a 20 mm carapace length as a cutoff for distinguishing juveniles from adults in the field based on previous data collected in Michigan (Budnick et al. 2022).

We included DNA from six other sympatric or geographically proximal crayfish species collected in Michigan ( *Creaserinus fodiens* (*N* = 3), *Cambarus thomai* ( *Lacunicambarus thomai* ) (*N* = 3), *Faxonius propinquus* (*N* = 1), *Faxonius virilis* (*N* = 1), *Lacunicambarus polychromatus* (*N* = 1), and *Procambarus acutus* (*N* = 1); Smith et al. 2019) in the study for genotyping to differentiate and eliminate any taxonomically misidentified or potentially hybrid samples using a principal component analysis (see below). Additionally, previously sequenced samples collected from Louisiana (*N* = 17) in the *P. clarkii* native range (Sard et al. 2023) were included for demographic analyses.

### 2.2 Library Preparation and Sequencing

Genomic DNA was extracted from gill tissue of adult crayfish. RAD capture libraries were prepared following the BestRAD protocol (Ali et al. 2016) using *SbfI* restriction enzyme (New England Biolabs). Capture reactions were carried out using the MyBaits Version 4.01(Daicel Arbor Biosciences; Ann Arbor, MI, USA). RAD baits were well distributed across chromosomes (see Figure S1 in Adams et al. 2024). For more information on the development and use of the RAD capture baits see Adams et al. (2024). The four capture libraries were pooled into one sequencing library that included equal DNA quantities from each of the capture libraries. The RAD capture library was then sequenced on one lane of Illumina's NovaSeq 6000 as 150 bp paired‐end reads at the Research Technology Support Facility (RTSF) Genomics Core at MSU. We created 36 total libraries for two independent sequencing runs. The first 12 were sequenced in 2021 and the remaining 24 were sequenced in 2022. These libraries included additional samples of *P. clarkii* for a previous project (Adams et al. 2024) that were simultaneously prepared and sequenced. For more detail on DNA extraction and library preparation see Data S1.

### 2.3 Sequence Processing

RAD capture sequence reads in the forward and reverse files for each library were exchanged whenever the barcode was found at the start of read two using a previously published perl script (bRAD_flip_trim.pl.; originally developed by Paul Hohenlohe, University of Idaho, and modified by Brian Hand and Seth Smith, University of Montana). Libraries were demultiplexed using process_radtags and PCR duplicates were removed using clone_filter in Stacks v. 2.59 (Catchen et al. 2013). Individual sequences after demultiplexing libraries are available on the NCBI Sequence Read Archive (Bioproject PRJNA1148680). Illumina adapter sequences and reads shorter than 50 bp were removed and reads were trimmed if the mean base quality dropped below Q15 using a sliding window of four bases using Trimmomatic v. 0.39 (Bolger et al. 2014). Reads were mapped to the *P. clarkii* reference genome (GCA_020424385.2; Xu et al. 2021) using BWA‐MEM v. 0.7.17 (Li and Durbin 2010; Li 2013). Mapped reads were sorted and indexed; then reads with mapping qualities < 20 and those not mapped in proper pairs were filtered out using SAMtools v. 1.9 (Li et al. 2009). Genotypes were then called using the gstacks module of Stacks v. 2.4 (Catchen et al. 2013; Rochette et al. 2019). To estimate measures of *P. clarkii* population genetic diversity, inter‐population genetic divergence, and gene flow, the SNP dataset started with 138,876 genotyped RAD loci, 521,238 variant loci, a mean insert length of 312.6 bp (±95.1 stdev), and a mean per‐sample coverage of 24.1× (±26.6 stdev).

We filtered genomic data to retain high‐quality samples and loci using VCFtools v. 0.1.15 (Danecek et al. 2011) and an iterative filtering approach (Figure S1). The final dataset excluded loci with quality scores less than 20, minor allele counts less than 3, and loci with greater than 55% missing data. We removed individuals with greater than 70% missing data and kept only one SNP per RAD tag, ensuring loci were separated by at least 100 kb on the reference genome. After filtering the dataset, 763 individuals and 2675 SNPs were used for the gene flow analyses (Table S2). For the genetic diversity statistics, after stricter filtering on SNP locus missingness, we retained 653 individuals and 1315 SNPs (Table S2). For more details on variant filtering, see Data S1. Samples from Louisiana, which is in the species' native range, had a greater proportion of missing genotypes but were necessary to include in our demographic analyses. Therefore, we created a second dataset that was processed as above except we removed loci with greater than 50% missing data, individuals with greater than 75% missing data, and loci with a minimum depth less than five.

### 2.4 Identifying Local Genetic Structure

Spatial population structure based on measures of inter‐waterbody variation in *P. clarkii* SNP allele frequency was analyzed using principal components analysis (PCA) as well as supervised and unsupervised clustering analyses. We conducted a PCA using Plink 2.0 v. 10.2 (Chang et al. 2015) and a discriminant analysis of principal components (DAPC) using the adegenet R package v. 2.1.7 (Jombart and Bateman 2008; Jombart and Ahmed 2011). Unsupervised clustering was done using the find.clusters function in adegenet, followed by a DAPC. To ensure we did not overfit the DAPC, we used the function optim.a.score to identify the optimal number of axes for retention in the PCA steps of the DAPC. We then re‐ran the DAPC with that optimal number of PC axes. We conducted DAPC analyses across all samples and within each genetically distinct geographic region separately. PCA ordination showed that two samples from Group 3 were intermediate between crayfish species; these samples were therefore removed from further analyses to avoid inclusion of possible interspecific hybrids (Figure S2).

For analyses based on populations rather than individuals, we removed any waterbody with fewer than five individuals (*N* = 3) for statistical purposes. We visualized genetic relationships among sampled locations using a neighbor‐joining tree (Saitou and Nei 1987). Bootstrap support for the nodes was calculated using the aboot function from poppr v. 2.9.3 (Kamvar et al. 2014, 2015). We visualized the tree with the plot.phylo function from the R package ape v. 5.6–2 (Paradis and Schliep 2019). Additionally, to minimize the effect of missing data on summary statistics, we removed loci and individuals that still had greater than 20% missing data using the R packages adegenet and poppr. We estimated the following genetic diversity summary statistics for *P. clarkii* samples in each waterbody: allelic richness (rarefied to 12 alleles), observed and expected heterozygosity, mean inbreeding coefficient (F_IS_; Nei 1987), and pairwise divergence in SNP allele frequency (F_ST_; Nei 1987) using the R package hierfstat v. 0.5–10 (Goudet 2005). The number of private alleles was enumerated using strataG v. 2.5.01 (Archer et al. 2017). We used Kruskal–Wallis rank sum tests with a Benjamini‐Hochberg multiple test correction to detect significant differences among geographically proximal and genetically similar groups for allelic richness, private alleles, heterozygosity, and F_IS_. The significance of F_ST_ was evaluated by calculating the 95% confidence interval of pairwise F_ST_ values between waterbodies using the boot.ppfst function (nboot = 1000) in hierfstat.

To test the hypothesis that genetic differentiation between *P. clarkii* individuals varied as a function of inter‐waterbody geographic distance (isolation‐by‐distance; Wright 1943), we conducted a matrix regression analysis (Mantel test) using linearized inter‐waterbody differentiation (*F*_ST_/1−*F*_ST_) versus the natural log of geographic distance following Rousset (1997). Pairwise geographic distances between sampling sites were calculated as great‐circle distances in meters in the geodist() function from the R package geodist v. 0.0.8 (Padgham 2021), and the R package vegan v. 2.6–2 (Oksanen et al. 2022) was used to conduct the Mantel test. Mantel tests were also conducted within Group 2 and Group 3 to look for isolation‐by‐distance within the geographic regions. The sample size of Group 1 was too small to confidently test for isolation‐by‐distance. The direction of gene flow was assessed using the directionality index, ψ, estimated by the R package rangeExpansion v. 0.0.0.9000 (Peter and Slatkin 2013). For the directionality index analysis, we removed waterbodies with fewer than five individuals.

### 2.5 Landscape Genetics Analysis

#### 2.5.1 Preparing Raster Layers

To test for effects of landscape features as barriers to gene flow among waterbodies, we tested a series of resistance surface models (Anderson et al. 2010; Spear et al. 2010). Modeled environmental features included canopy cover, land cover, roads, and hydrography, which were chosen based on organismal and waterbody ecology as well as data availability. Canopy cover and land use data were drawn from the National Land Cover Database (NLCD; Dewitz 2021). Hydrography data were obtained from the National Hydrography Dataset (U.S.Geological Survey 2023) and road data, including traffic volumes, were obtained from the Michigan GIS Open Data Portal (Michigan Department of Transportation 2023). Roads were ranked as low traffic (< 10,000 vehicles/24 h), medium traffic (10,000–50,000 vehicles/24 h), or heavy traffic (> 50,000 vehicles/24 h). The nine NLCD land cover categories that were present in our study areas were collapsed into seven categories by combining deciduous forest and mixed forest as well as pasture/hay and cultivated crops, leaving remaining categories of open space development, low intensity development, medium intensity development, high intensity development, and woody wetlands. Hydrography was represented as the presence or absence of water features and canopy cover was a continuous surface representing percent coverage. As line features, the roads and hydrography datasets were converted to polygons using a 60 m buffer before conversion to a 30 m resolution raster that was subsequently aligned to the 30 m resolution NLCD and canopy cover layers. Raster processing was conducted in ArcGIS Pro 3.1.2 (ESRI).

#### 2.5.2 Running Resistance Analyses

We estimated inter‐waterbody resistance distances (*sensu* Spear et al. 2010) using the R package ResistanceGA v. 4.2–9 (Peterman 2018). The resistance distance measures the difficulty of movement between waterbodies across the landscape, based on how the chosen landscape features—land cover types, water connectivity, etc.—facilitate or impede gene flow. This allows us to test whether landscape resistance better explains genetic differentiation than simple geographic distance alone. The resistance distances were estimated using CIRCUITSCAPE v. 5.13.1 (McRae 2006; McRae et al. 2008) written in the Julia programming language v. 1.9.3 (https://julialang.org/). Using circuit theory, CIRCUITSCAPE calculates the effective resistance between waterbodies, treating the landscape like an electrical circuit where the flow of current represents potential gene flow and resistance represents barriers weighted by the extent to which they impede gene flow. We used the GA.prep and jl.prep functions in ResistanceGA with default parameters and our F_ST_ matrix. We ran single surface optimization for each of our variables of interest and multi‐surface optimization for all our variables and for the following combinations: canopy cover + hydrography + roads, canopy + roads, canopy + hydrography, hydrography + roads. In addition, ResistanceGA evaluates a model of isolation‐by‐distance and a null model with only the intercept. The best optimization of the resistance surface was chosen based on Akaike's Information Criterion adjusted for small‐sample sizes (AICc, Burnham and Anderson 2004). We then conducted a bootstrap analysis using the resist.boot function to assess the relative support for the optimized resistance surfaces. Models within two AIC units (ΔAICc ≤ 2) of the best‐supported model were considered well supported (Row et al. 2017).

#### 2.5.3 Demographic Modeling

**FIGURE 2:**
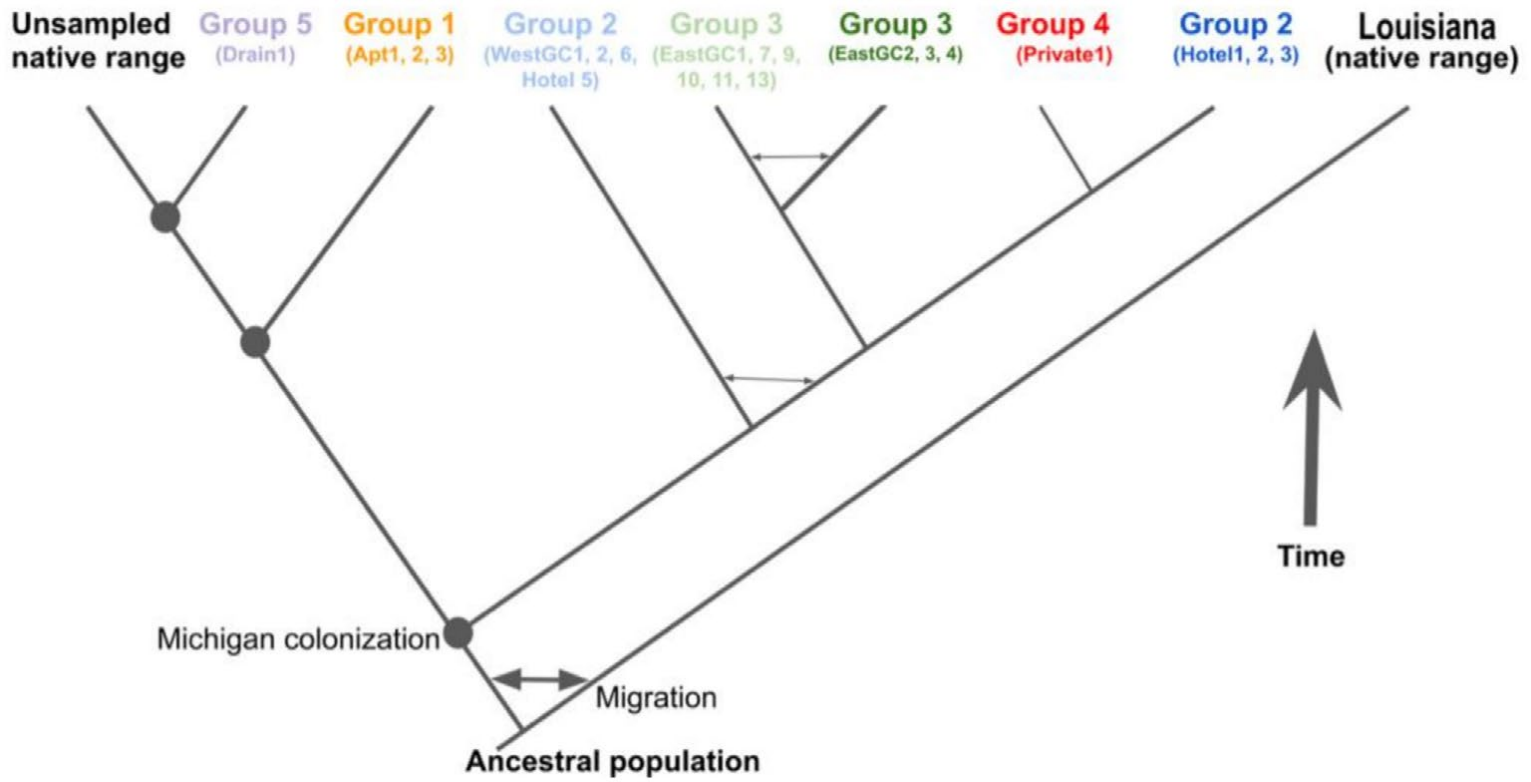
Schematic of one tested coalescent demographic model structure used to simulate the colonization history and demographic history of southeastern Michigan *P. clarkii* populations. The width of the arrows within the tree indicates the hypothesized relative strength of migration. Circles indicate the hypothesized introduction events. This tree represents the best supported model which includes colonization of Group 2, followed by a bridgehead effect and an independent colonization of Group 1 and Group 5 (Grp2_bridge_indGrp1_5). See Figure S3 for the other nine models tested.

For the demographic modeling, our dataset started with 190,442 genotyped loci, 974,456 variant loci, a mean insert length of 312.9 bp (±95.7 stdev), and a mean effective per‐sample coverage of 23.8× (±26.6). After filtering the dataset, 948 individuals and 1808 SNPs remained for the demographic analyses (Table S2). We used Approximate Bayesian Computation (ABC) to reconstruct the invasion history of *P. clarkii* in SE Michigan by comparing ten competing demographic models (Figure 2, Figure S3). These models tested two main dispersal hypotheses: stepping‐stone (dispersal between adjacent regions) and bridgehead (initial site becoming source of multiple secondary introductions). In our analyses, support for a bridgehead effect model could indicate human‐assisted movement between regions, whereas support for a stepping‐stone model would indicate natural dispersal and range expansion following the initial invasion of the species. The models focused on Groups 2 and 3 as potential secondary sources, based on initial reports and population sizes (Smith et al. 2018; Sard et al. 2023). We used coalescent simulations in fastsimcoal2 v. 2.709 (Excoffier et al. 2021) using modified wrapper functions from the strataG R package (v 2.5.5 Archer et al. 2017), assuming a one‐year generation time (Huner and Barr 1991), a mutation rate of 3.6 × 10^−9^ (Liu et al. 2016), and a 150 bp sequence length (see Table S3 for additional prior values). Model selection was performed using multinomial logistic regression, neural network, and random forest methods, with parameter estimation (effective population size and bottleneck severity) conducted for the best‐supported models. Previous estimates of effective population size from multiple waterbodies in this region ranged from 18 to 250 (see Table 1 in Adams et al. 2024). Leave‐one‐out cross validations were used to assess the level of confidence in results from the model selection and parameter estimation analyses. For details on models, simulations, summary statistics, and ABC analyses see Data S1.

**TABLE 1:** Measures of genetic diversity and sample size (*N*) for individuals sampled from 20 invasive *P. clarkii* collection locations in the Detroit metropolitan area in SE Michigan, including allelic richness (*A*_r_), the number of private alleles (*A*_p_), mean observed heterozygosity (*H*_o_), mean expected heterozygosity (*H*_e_), and inbreeding coefficient (*F*_IS_). Groups are based on levels of genetic differentiation and geographic proximity (see Figure 1). The number of females (F) and males (M) in each waterbody is indicated next to the total sample size. Full names of waterbodies can be found in Table S1.

Group	Waterbody	*N* (F, M)	*A*_r_	*A*_p_	*H*_o_	*H*_e_	*F*_IS_
1	Apt1	24 (10, 14)	1.247	24	0.081	0.088	0.079
	Apt 2	6 (2, 4)	1.427	1	0.061	0.07	0.095
	Apt 3	6 (1, 5)	1.302	0	0.072	0.076	0.038
2	WestGC1	69 (36, 33)	1.099	0	0.03	0.031	0.052
	WestGC2	33 (15, 18)	1.102	0	0.029	0.032	0.056
	WestGC6	18 (7, 11)	1.121	1	0.036	0.039	0.049
	Hotel1	108 (49, 59)	1.046	1	0.017	0.018	0.076
	Hotel2	11 (4, 7)	1.042	0	0.016	0.016	0.003
	Hotel3	27 (16, 11)	1.047	0	0.016	0.018	0.080
	Hotel4	30 (10, 20)	1.044	0	0.016	0.017	0.029
3	EastGC1	8 (6, 2)	1.115	0	0.031	0.034	0.042
	EastGC2	69 (43, 26)	1.085	0	0.026	0.028	0.064
	EastGC3	9 (5, 4)	1.111	0	0.029	0.03	0.027
	EastGC4	51 (27, 24)	1.085	0	0.027	0.028	0.042
	EastGC7	28 (20, 8)	1.104	0	0.029	0.031	0.058
	EastGC10	33 (14, 19)	1.081	0	0.022	0.025	0.100
	EastGC11	27 (14, 13)	1.087	0	0.026	0.028	0.073
	EastGC13	35 (21, 14)	1.098	0	0.031	0.031	0.033
4	Private1	25 (8, 17)	1.069	32	0.028	0.028	−0.017
5	Drain1	36 (22, 14)	1.532	647	0.147	0.161	0.070

## 3 Results

### 3.1 Population Structure and Genetic Diversity

**FIGURE 3:**
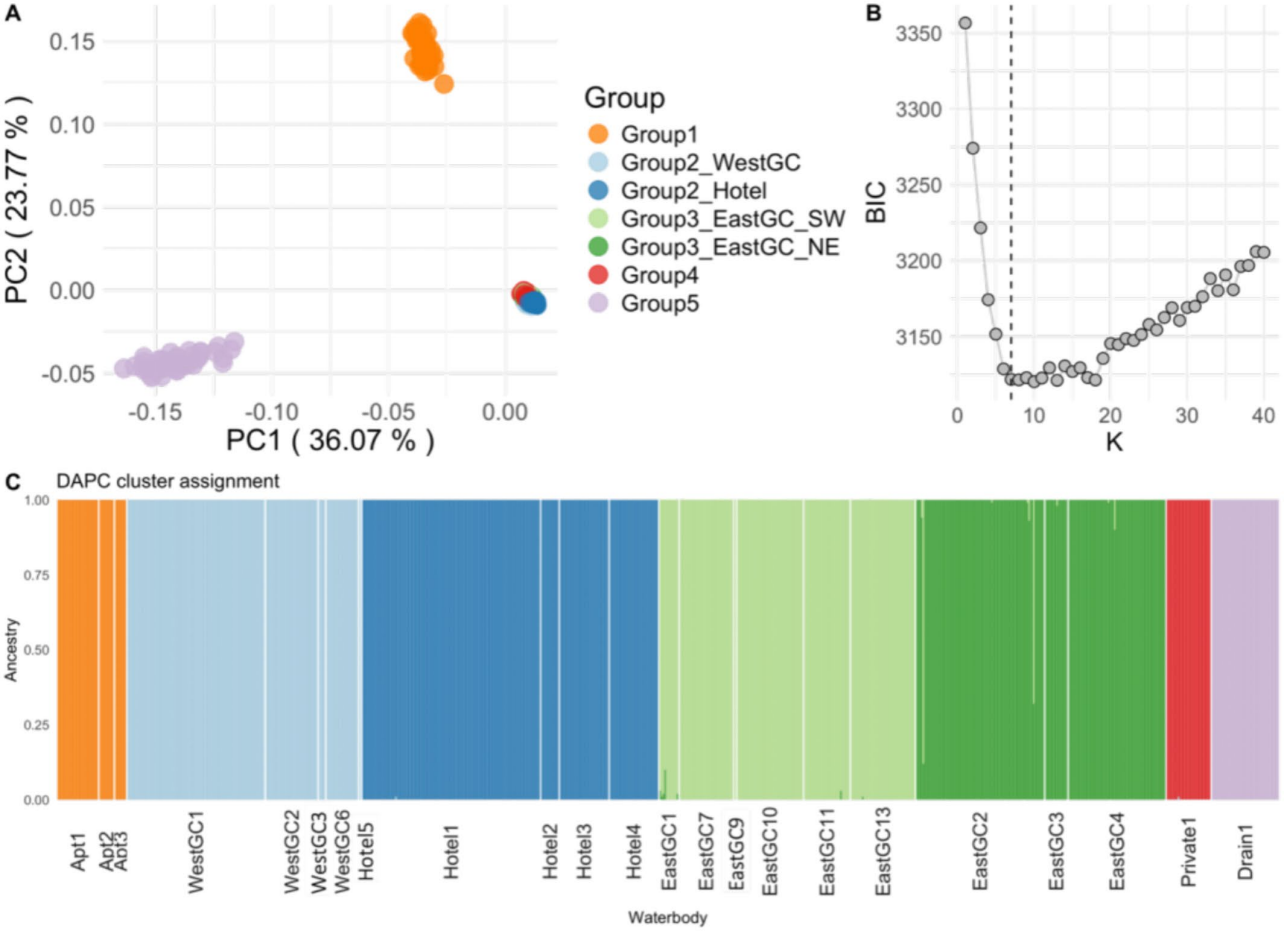
(A) Principal components analysis and (B) discriminant analysis of principal components (DAPC) cluster analysis showing the Bayesian Information Criterion (BIC) support for successive numbers of clusters (K) consistent with the multi‐locus SNP genotype data. The dashed vertical line indicates the seven clusters we chose. (C) Bar plot showing the distinct genetic groups (by group color) within the study area. Analyses are based on 2675 SNPs and 763 individuals.

**FIGURE 4:**
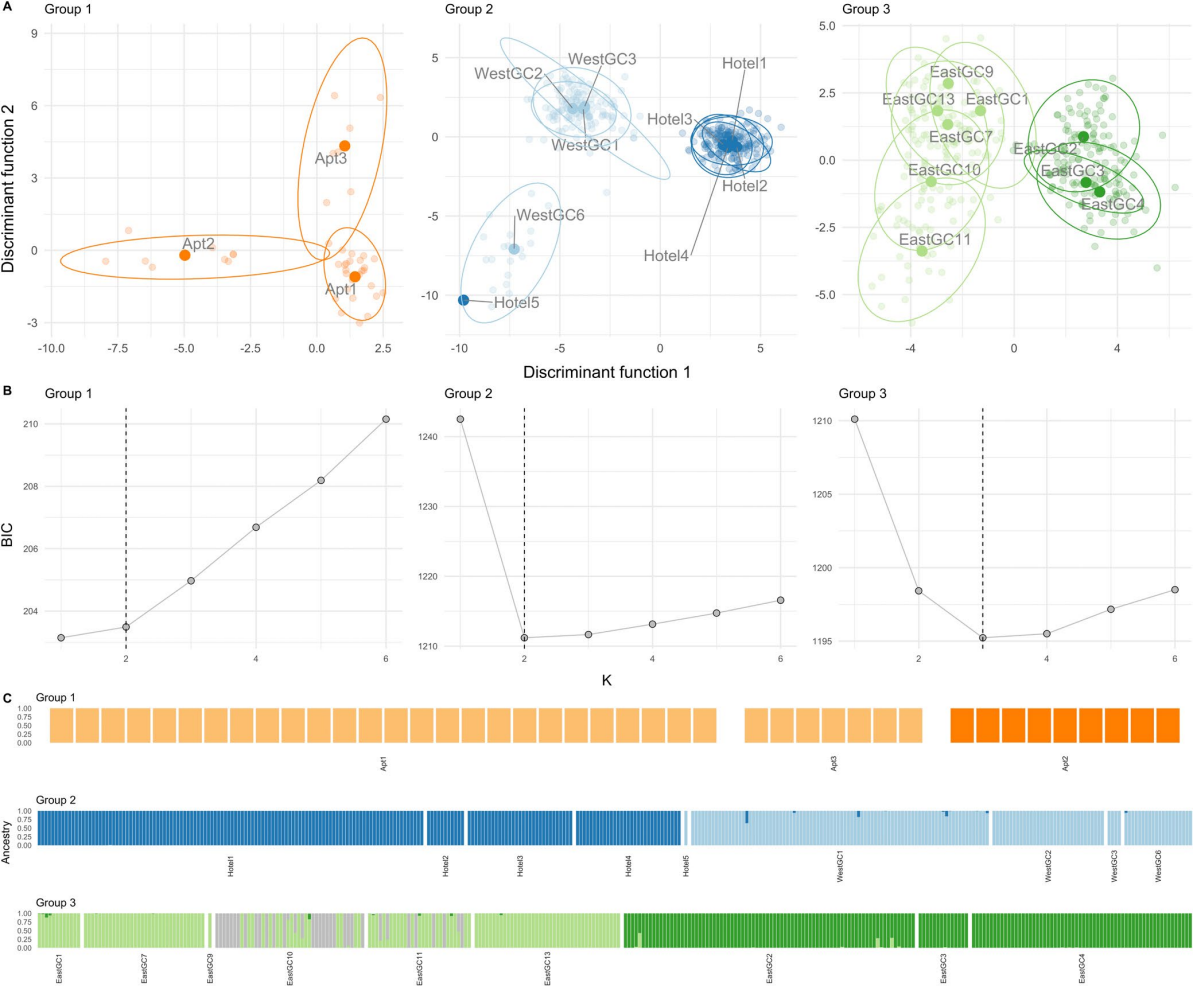
(A) Individual ordination based on discriminant analysis of principal components (DAPC) showing discriminant function two versus one. (B) DAPC cluster analysis for geographic Groups 1, 2, and 3 showing the Bayesian Information Criterion (BIC) for successive numbers of clusters (K) best supported by SNP genotype data. The dashed vertical line indicates the number of clusters chosen to show in the bar plots, typically the K with the lowest BIC. (C) Bar plots showing the proportion of ancestry assigned to a specific ancestry cluster based on the best K for each individual. Note that the ‘best K’ for Group 1 is one, but we present two clusters in the bar plot. Individuals are aggregated by waterbody within each group. On the basis of 2675 SNPs and the following sample sizes: Group 1 *N* = 42, Group 2 *N* = 333, Group 3 *N* = 317.

The principal component analysis and unsupervised clustering analyses indicated hierarchical clustering patterns that corresponded closely with geographic distribution across the study area (Figures 3, 4). In the PCA, samples from Group 5 separated from Groups 2, 3, and 4 along component one, which represented 36% of the variance (Figure 3A). Samples from Group 1 separated from Groups 2, 3, and 4 along component two, which represented almost 24% of the variance (Figure 3A). A discriminant analysis of principal components (DAPC) across all samples suggested that between seven and nine clusters best explained the data based on the Bayesian Information Criterion (BIC) values (Figure 3B). When the proportion of ancestry associated with each genetic cluster for each individual was visualized, we identified seven genetically distinct groups that were largely differentiated according to geographic location (Figure 3C), with two additional genetic clusters within Groups 2 and 3 (Figures 3C, 4). When we repeated the DAPC analysis within Groups 1, 2, and 3 separately, the two genetic clusters within Groups 2 and 3 were visually supported (Figure 4A–C). Within Group 2, samples segregated into two genetic clusters along the northwest‐southeast diagonal (Figures 1, 4). We identified a similar geographic pattern within Group 3 in which samples from the southwest portion of the study area were assigned to the same cluster while samples from waterbodies in the northeast portion of the study area were assigned to a different cluster (Figures 1, 4). We also identified samples in the southern‐most waterbodies assigned to a third cluster within Group 3, but were interspersed with the dominant cluster in that geographic region (Figure 4C). Although we show that Group 1 can be separated into two clusters when evaluated alone, one cluster best describes those samples based on the lowest BIC (Figure 4). Groups 4 and 5 only included one waterbody and one genetic group each, so DAPC analyses were not conducted separately.

The neighbor‐joining tree topology showed the same major patterns as the PCA and clustering analyses including multiple well‐supported distinct genetic clusters. Specifically, Group1 appeared more distantly genetically related to the remaining groups as indicated by tree topological location and long branch lengths. Two genetic clusters within Groups 2 and 3 were also apparent (Figure S4).

Genetic diversity measures varied substantially among geographic groups, with Groups 1 and 5 showing the highest diversity levels (Table 1). Group 5 had the highest allelic richness (*A*_r_ = 1.16), number of private alleles (*A*_p_ = 647), and observed and expected heterozygosity (*H*_o_ = 0.15, *H*_e_ = 0.16, respectively; Table 1). The number of private alleles in Group 5 comprised the vast majority of all those documented. Group 2, specifically the northeast waterbodies that include the pond at Hotel1, had the lowest allelic richness and observed and expected heterozygosity (Table 1). Most waterbodies showed similar genetic diversity between sexes, consistent with the XX/XY sex determination system in *P. clarkii* (Shen et al. 2022), with the notable exception of a potential female bias of private alleles in one waterbody in Group 1 (Table S4). The inbreeding coefficient, *F*_IS_, ranged from −0.02 in Group 4 to 0.095 in one waterbody in Group 1. *F*_IS_ was positive in samples from 19 of 20 waterbodies indicating a deficiency of observed relative to expected heterozygosity, under Hardy–Weinberg equilibrium (Table S5). F_IS_ was significantly different from zero in 14 out of 20 of the sampled waterbodies, and all of the significant values were positive (Table S5). After grouping waterbodies into geographically proximal and genetically similar groups, retaining two groups within Group 2 and 3 each based on unsupervised clustering (Figures 3, 4), we found no significant differences in the number of private alleles (*H*_6_ = 11.8, *p* = 0.066) or the inbreeding coefficient (*H*_6_ = 5.04, *p* = 0.539) among groups. Significant differences were found between groups for allelic richness (*H*_6_ = 16.59, *p* = 0.011), observed heterozygosity (*H*_6_ = 15.96, *p* = 0.014), and expected heterozygosity (*H*_6_ = 16.74, *p* = 0.01). However, none of the pairwise comparisons were significant after a Benjamini–Hochberg multiple test correction for these three measures.

**FIGURE 5:**
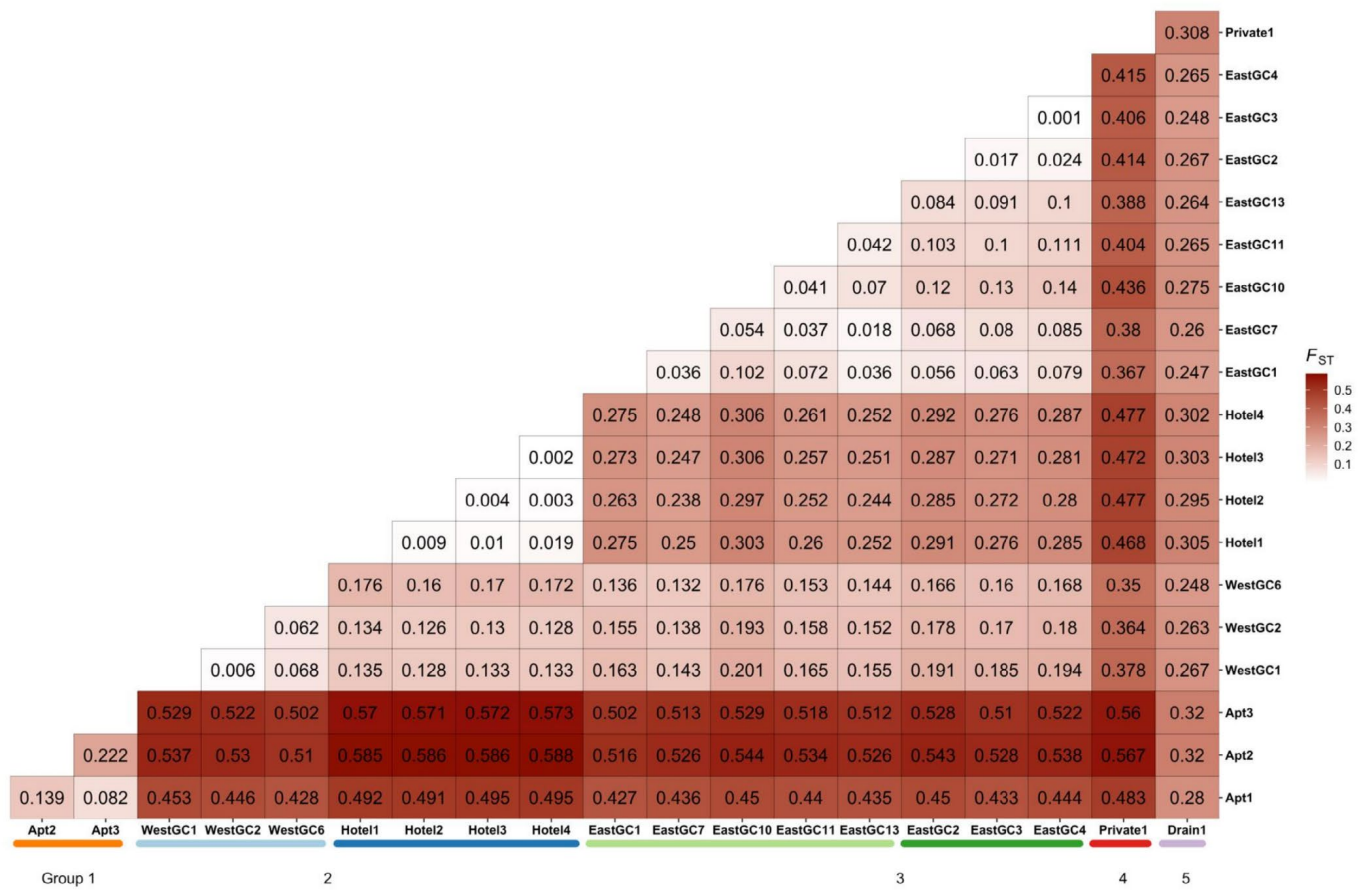
Pairwise *F*_ST_ estimates (Nei 1987) for *P. clarkii* sampled from 20 waterbodies. Groups are indicated with colored bars underneath the waterbody labels. Estimates are based on 1315 SNPs and 653 individuals.

Pairwise *F*_ST_ values supported the geographic clustering patterns, showing strong genetic differentiation among groups and variable levels of divergence within groups. The overall average between waterbody population *F*_ST_ was 0.279. The range was from 0.001 between two northeast waterbodies in Group 3 (EastGC3 and EastGC4) to 0.588 between a waterbody in Group 2 (Hotel4) and one in Group 1 (Apt2; Figure 5). Group 1 was the most differentiated from Groups 2, 3, and 4 with *F*_ST_ values ranging from 0.427 to 0.588 (Figure 5). Within geographic groups containing multiple waterbodies, the average between waterbody population *F*_ST_ values was highest within Group 1 (0.148) and lowest within Group 3 (0.07). Most pairwise *F*_ST_ values were significantly different from zero except for all values between the northeast waterbodies within Group 2 and between the two northeast ponds in Group 3 (EastGC3 and EastGC4).

**FIGURE 6:**
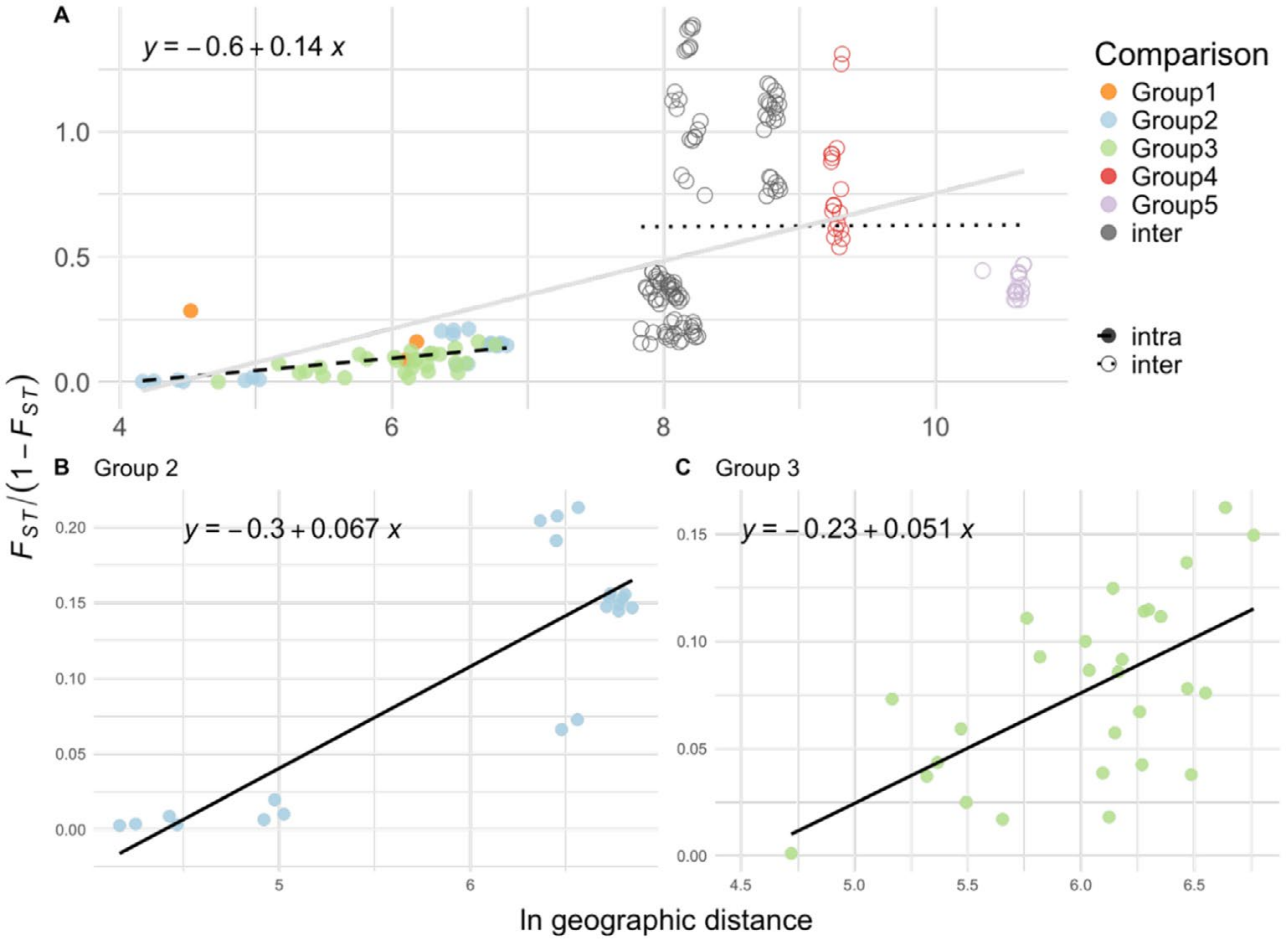
Pairwise genetic differentiation (linearized *F*_ST_) between *P. clarkii* from waterbodies in southeastern Michigan. Estimates of pairwise differentiation are plotted against the natural logarithm of geographic distances (Rousset 1997) for (A) all groups, (B) Group 2, and (C) Group 3. Linear regressions are shown as solid lines as well as their equations for all groups, Group 2, and Group 3. Their *R* ^2^ values are 0.25, 0.75, and 0.36, respectively. Pairwise comparisons are divided into intra‐ (solid circles) and inter‐group (open circles). Linear regressions are plotted for intra‐ (dashed) and inter‐group (dotted) comparisons as well; their equations are *y* = −0.2 + 0.049*x* (*R* ^2^ = 0.31), *y* = 0.6 + 0.0027*x* (*R* ^2^ = 4 × 10^−5^), respectively. Analyses are based on 1315 SNPs from 653 individuals, 296 individuals for Group 2, and 260 individuals for Group 3.

Genetic differentiation among waterbodies showed a significant isolation‐by‐distance pattern across the study area and within major population groups. Using the Mantel tests to describe correlations between geographic distance (km) and genetic differentiation (*F*_ST_) among waterbody populations, we detected a significant association indicating a pattern of isolation‐by‐distance across all samples (*r* = 0.504, *p* = 0.003; Figure 6A), as well as between waterbodies within Group 2 (*r* = 0.864, *p* = 0.001; Figure 6B) and Group 3 (*r* = 0.597, *p* = 0.002; Figure 6C). The other groups had too few waterbodies to allow analyses to be performed. The broad IBD pattern detected is likely an artifact of multiple genetically divergent introductions that are also geographically separated, instead of a signal of natural gene flow across the entire region. We observed low inter‐waterbody connectivity, based on the directionality index (Ѱ), between Groups 1 and 5 and the rest of the groups (Figure S5A). The directionality index ranged from −0.653 to 0.789 across all pairwise comparisons. All z‐scores greater than two (*N* = 15) were detected in comparisons between Group 5 and Groups 2, 3, and 4. Looking within groups, the values of the directionality index were relatively lower, ranging from −0.139 to 0 in Group 1, −0.381 to 0.092 in Group 2, and −0.145 to 0.102 in Group 3 (Figure S5B–D).

Landscape genetic modeling revealed that isolation‐by‐distance alone best explained genetic differentiation patterns, with landscape features showing no significant influence on gene flow. For both Group 2 and Group 3, IBD was the best‐supported model to explain our inter‐waterbody genetic differentiation across the landscapes (Group 2 average AICc = −29.08, Group 3 average AICc = −52.96; Table S6). None of our landscape variables (canopy cover, hydrography, land cover, or roads) were identified as significant factors contributing to inter‐waterbody F_ST_ in any group evaluated. Despite high‐traffic highways between waterbodies in Group 2, roads appeared to have lower resistance than other landscape features (Figure S6A). In our results from Group 3, the model with only roads was second best (AICc = −45.32) despite contributing little to the composite optimized surface (Figure S6B). The ΔAICc for the roads model was greater than two suggesting IBD was singularly supported.

### 3.2 Demographic Modeling

Demographic modeling consistently supported independent introductions for Groups 1 and 5 (cumulative posterior probability ranging 0.46–0.95 across methods; Table 2), while providing mixed support for stepping‐stone versus bridgehead invasion models among the remaining groups. The most supported model was the stepping‐stone model starting from Group 3 with independent Group 1 and Group 5 introductions based on the rejection and neural network methods (Table 2). The bridgehead model starting from Group 2 with independent Group 1 and Group 5 introductions had the most support based on the multinomial logistic regression and random forest approaches (Table 2). Despite these “best” models, we found fairly even support for the stepping‐stone and bridgehead models and for the initial invasion in Group 2 and Group 3, when these models also included independent Group 1 and Group 5 introductions. Collectively these four models represent between 88% and 95% of the posterior support from the multinomial logistic regression approach, 920 of the 1000 trees in the random forest, and 59%–72% of the posterior support from the neural networks (with remaining support evenly distributed across other models).

**TABLE 2:** Posterior probabilities from ABC model selection based on four algorithms: (A) rejection, multinomial logistic regression (MN log), neural network, and (B) random forest. The number of accepted replicates is shown for each of the three tolerances (Tol). In (B) the number of decision trees that support each model is shown under each model. The most supported model for each combination of model and tolerance is shown in bold. Models with secondary spread starting from Group 2 are indicated with “Grp2” and from Group 3 as “Grp3”. “Step” indicates the models based on stepping‐stone versus bridgehead (“bridge”) models. Models that had an independent Group 1 and Group 5 introduction are indicated with “indGrp1_5”. The model with independent colonizations for Groups 1, 2, 3, 4, and 5 is labeled “5cols”. The model of secondary spread from west to east is labeled as “W2E”. For additional model details see Figure S3.

Algorithm	Tol	Accepted reps	5 cols step	Grp3_bridge	Grp3_bridge_indGrp1_5	Grp3_step_5	Grp3_step_indGrp1_5	Grp2_bridge	Grp2_bridge_indGrp1_5	Grp2_step	Grp2_step_indGrp1_5	W2E step
**(A)**												
Rejection	0.005	5000	0.063	0.0944	0.1334	0.1026	0.1338	0.0862	**0.1354**	0.0798	0.117	0.0544
	0.01	10,000	0.0698	0.0975	0.1279	0.1098	**0.1319**	0.0928	0.1231	0.0756	0.1136	0.058
	0.025	25,000	0.0800	0.1028	0.1206	0.1116	**0.1293**	0.0982	0.1078	0.0766	0.1068	0.0664
MN log	0.005	5000	0.0197	0.0065	0.3159	0.0123	0.1621	0.0052	**0.3603**	0.0041	0.1099	0.0042
	0.01	10,000	0.0389	0.0045	0.1414	0.0034	0.2015	0.0034	**0.3837**	0.0052	0.1655	0.0526
	0.025	25,000	0.0248	0.0132	**0.2538**	0.0171	0.1899	0.0368	0.2361	0.0098	0.2047	0.0139
Neural network	0.005	5000	0.0485	0.0705	0.1282	0.0796	0.1387	0.0764	**0.1717**	0.0743	0.152	0.0600
	0.01	10,000	0.0566	0.0845	0.1749	0.0695	**0.1819**	0.0600	0.1479	0.0439	0.1316	0.0494
	0.025	25,000	0.0564	0.0735	0.1998	0.0406	**0.2153**	0.0414	0.1493	0.0213	0.1553	0.0471
**(B)**												
**Algorithm**	**Best model**	**Posterior Prob**.										
Random forest	Grp2_bridge_indGrp1	0.6286	8	8	219	0	110	6	**404**	2	187	56

Cross‐validation analyses confirmed the reliability of model selection methods, with multinomial logistic regression and random forest approaches showing the highest accuracy in identifying correct demographic models. The multinomial logistic regression showed higher posterior probabilities for the true model during cross‐validation (Figure S7), indicating greater confidence in correct model identification compared to neural networks. The random forest approach had an overall 77% accuracy with low out‐of‐bag error (23.24%), as shown in the confusion matrix (Table S7). The models with the highest out‐of‐bag errors in the random forest confusion matrix were bridgehead models with independent Group 1 and Group 5 introductions starting in both Group 2 (error = 0.344) and Group 3 (error = 0.325; Table S7). These models were most often confused with stepping‐stone models from the same group (34% of erroneous classifications), while the Group 3 bridgehead model showed more distributed misclassification patterns, with 24% confused with the corresponding stepping‐stone model.

Parameter posterior distributions estimated via ABC resembled prior distributions in all cases (Figure S8), although with slight differences in posterior density for effective population size parameters between groups (Table S8). This pattern suggests limited ability to estimate model parameters, given the summary statistics for genetic diversity and differentiation used in our analysis. Cross‐validation results also support this conclusion, particularly for migration and bottleneck severity parameters (Figure S9). Given these observations, we focus our discussion of demographic modeling results below on our model selection describing models of best fit characterizing founding history and secondary gene flow, rather than parameter estimation.

## 4 Discussion

Aquatic resource managers routinely utilize population genetic data and computational methods in the management and control of aquatic invasive species (Darling and Mahon 2011; Bernos et al. 2022). Importantly, in the context of the present study, genomic data and analyses allow managers to reconstruct the invasion histories of aquatic invasive species, inferring the most likely source populations and dispersal routes (Wellband et al. 2017; Resh et al. 2021).

The *P. clarkii* invasion of Michigan has proceeded through a combination of multiple introductions across the 153km^2^ study area surveyed and secondary spread on smaller spatial scales. We detected multiple genetic clusters in a relatively small geographic area. Genetic analyses suggest that there is minimal gene flow between geographically proximal groups of *P. clarkii‐infested* waterbodies, but within groups, genetic differentiation among waterbodies fits a pattern of isolation‐by‐distance. Demographic analyses indicate minimally three separate introductions in SE Michigan, as hypothesized in previous work (Sard et al. 2023). These findings suggest that effective management efforts must include controlling established local populations (particularly if vulnerable habitats or native populations are nearby), and sustained efforts to further identify and prohibit long‐distance vectors that have led to repeated introductions from the native range. This study adds to the growing body of literature showing that genetic tools are valuable in understanding the colonization and subsequent dispersal in invasive species (reviewed in Mcgaughran et al. 2024).

### 4.1 Genetic Structure Suggests Multiple Introduction Sources

Genomic data and analyses provide a means of reconstructing invasion histories of *P. clarkii* in SE Michigan, and a source of inference of the most likely number of source populations and post‐colonization dispersal routes. Ordination and unsupervised clustering analyses supported three major genetic clusters in our *P. clarkii* samples: Group 1, Group 5, and Groups 2, 3, and 4. Since these populations of *P. clarkii* were only discovered in 2017 (Smith et al. 2018), such high genetic divergence suggests that these clusters are due to multiple independent introductions, which aligns with previous evidence that there were multiple introductions in Michigan statewide as well as within the Detroit metropolitan area (Sard et al. 2023). The mechanism by which these genetic groups came about is still unknown but could be due to a combination of movement from pet and live food trades, bait shops, fisheries enhancement, and live specimens from biological supply companies for local classrooms (Hobbs III et al. 1989; Chucholl 2013; Smith et al. 2018; Oficialdegui et al. 2020; Alvanou et al. 2024; Olden and Carvalho 2024). Studies on invasive populations of *P. clarkii* elsewhere have also found strong spatial genetic structure (Barbaresi et al. 2007; Li et al. 2012; Huang et al. 2017; Yi et al. 2018; Bélouard et al. 2019; Acevedo‐Limón et al. 2020; Scoparo et al. 2023; Kendrick et al. 2024). Many have also attributed this pattern to multiple introductions from separate sources (Li et al. 2012; Paulson and Martin 2014; Huang et al. 2017; Yi et al. 2018; Acevedo‐Limón et al. 2020; Scoparo et al. 2023; Kendrick et al. 2024).

### 4.2 Fine‐Scale Genetic Structure and Limited, but Consistent, Secondary Dispersal

We detected genetic substructuring across Groups 2, 3, and 4 and especially within Groups 2 and 3, which showed pronounced genetic differentiation at small geographic scales (~1 km). Water bodies within Group 2 and Group 3 consisted of two genetic clusters each, both with axes of differentiation oriented in a northeast by southwest direction (Figure 1). Between the waterbodies of each cluster there are pronounced areas of extensively developed land, including heavily trafficked highways in Group 2 and a permanent stream in Group 3. One waterbody in Group 2 (Hotel5) located in the northeast of this geographic group was more genetically similar to the waterbodies in the southwest (Figures 3, 4), although more samples are needed to confirm this result. Sard et al. (2023) found a smaller genetic difference between waterbodies in the north versus the south of the golf course (0.061) in what we referred to as Group 3 than our estimate (~0.1), which is likely due to their low number of individuals. We cannot rule out that the difference could be due to the different RAD methods between the studies (RADseq versus RAD capture), which could result in different markers. However, RAD capture baits used in this study were developed from the Sard et al. (2023) data, so the impact is likely less than that of differences in the number of individuals between the two studies. Interestingly the waterbodies in the northeast in Group 3 drain to the north and a drainage ditch flows east, while the rest of the Group 3 waterbodies follow the creek drainage. This hydrological pattern could reinforce locations of the genetic clusters within Group 3. This pattern could also be due to multiple founding sources that were genetically more similar than the founders of Groups 1 and 5. Alternatively, strong bottlenecks due to multiple founding events from a single source could have also put these populations on divergent evolutionary trajectories early on. Genetic diversity measures support the latter explanation. Previous studies of *P. clarkii* in different invasion fronts have also suggested that the genetic divergence observed across introduced populations was partially due to strong founder effects and multiple introductions (Yue et al. 2010; Paulson and Martin 2014).

Human‐mediated “jump” dispersal at larger geographic scales in combination with natural secondary spread was detected in the study area. Strong spatial genetic structure, as evidenced by high *F*_ST_ values, suggests that there has been minimal gene flow between the water bodies associated with the different geographic groups in SE Michigan. Results are consistent with previous work that showed low gene flow in *P. clarkii* invasion fronts in North and South Carolina, USA (Kendrick et al. 2024), France (Almerão et al. 2018; Bélouard et al. 2019), and China (Huang et al. 2017; Yi et al. 2018). However, significant Mantel tests consistently documented associations between inter‐population genetic distance and geographic distance (isolation by distance; IBD) across genetic groups and our landscape genetic analyses indicate that some dispersal occurred, especially at finer geographic scales. Gene flow in our study area was directly observed based on pedigree analyses that documented siblings in multiple waterbodies (Adams et al. 2024). The patterns of IBD in Groups 2 and 3 are fairly consistent in that the slopes of the linear regressions were similar (0.067 and 0.051 for Groups 2 and 3, respectively), despite the distinct landscapes separating water bodies within these two regions. We found no evidence for separate colonization sources within these groups. Thus, local dispersal appears to be a factor in post‐colonization spread. Studies have shown that *P. clarkii* can easily move overland at distances ranging from tens of meters per day (Gherardi et al. 2002; Barbaresi et al. 2004; Aquiloni et al. 2005) to four kilometers per day in rice fields (Barbaresi et al. 2007). Independent studies in the study region have documented that *P. clarkii* has moved up to 100.18 m per hour in the water (Raboin et al. 2025). Further, our population and landscape genetics analyses suggest that developed land may not be acting as a dispersal barrier as previously believed. For example, in Group 2 three southwest waterbodies are on the West Golf Course and one genetically similar waterbody (WestGC6) is separated from them by paved roads and a large parking lot. Notably, there is a culvert under this developed land connecting these two areas, potentially contributing to gene flow and inter‐population genetic similarity (see Figure S6A). *P. clarkii* have been shown previously to use waterways for dispersal following colonization (Kerby et al. 2005; Paulson and Martin 2014; Bélouard et al. 2019). Although samples currently analyzed do not indicate gene flow is occurring between geographic groups, the IBD patterns detected among water bodies in close proximity and associated with different landscape features indicate that there is limited impedance to future dispersal across SE Michigan.

### 4.3 Demographic Modeling Supports Minimally Three Introduction Sources

Spatial genetic results are further supported by demographic modeling that indicated at least three independent introductions into the study area. An independent introduction of crayfish in geographic Groups 1 and 5 was consistently supported across the models evaluated. The best supported model using multinomial logistic regression suggested an initial introduction into water bodies associated with geographic Group 2, with subsequent colonization from Group 2 into Groups 3 and 4 (i.e., a bridgehead model of secondary colonization). However, alternative ABC model selection algorithms (neural networks and random forest) favored initial colonization in Group 3. While all approaches consistently supported independent introductions for Groups 1 and 5, we could not definitively resolve whether the primary invasion location was Group 2 or Group 3, or whether subsequent spread followed bridgehead or stepping‐stone patterns, so the mechanism of dispersal remains unresolved. We were also unable to confidently provide estimates of demographic parameters such as effective population sizes, bottleneck severity, or timing of colonization events. The recency of the SE Michigan invasion makes this system challenging due to the limited time for population divergence in allele frequency and potentially due to the strength of the founding bottlenecks. Future demographic modeling in this system may wish to explore alternative models contrasting different independent SE Michigan introductions and different rates of secondary spread.

### 4.4 Future Directions

While the results of this study point to two or three independent sources, the origins of these sources remain unknown. Possession of live *P. clarkii* has been prohibited since 2014 in the state of Michigan (Smith et al. 2018). However, the species can still be found for sale in pet stores and food markets in the state (Smith et al. 2018), although rare, and is widely accessible through online marketplaces (Olden and Carvalho 2024). An introduced *P. clarkii* population in western Michigan appeared genetically similar to samples collected from the native range, and (less so) to samples collected from a biological supply company (Sard et al. 2023). To better understand the sources of the introduced populations in SE Michigan, incorporating samples from potential origins into genetic structure and demographic modeling analyses would be beneficial. Identifying the sources of these introductions could help inform regulatory efforts especially in the metropolitan Detroit area.

Group 5 consistently deviated from the rest of the groups in many of our analyses with higher measures of genetic diversity. We did not find any samples from Group 5 that appeared to be misidentified as another species based on our ordination with the other native Michigan crayfish. Group 5 consisted of one sampled waterbody that was detected in 2019 2 years after the first infestation was detected in the region. It is interesting that Group 5 samples contained the most private allele diversity across all of the samples surveyed and the highest divergence. This pattern could be due to multiple introductions from different genetic sources into the same waterbody, or from a single introduction of a large number of founders that were genetically distinct from the others. Further, it could be that additional species or different sources of *P. clarkii* need to be included to understand the origins of Group 5, considering it is also geographically more distant than the other populations included in this study.

Our landscape genetic analyses were designed to test the hypothesis that certain physical landscape features such as landcover types, hydrological connectivity, and barriers such as roads may either facilitate or impede post‐colonization expansion. Lack of statistically supported parameter estimates from demographic modeling and our inability to establish associations between measures of genetic differentiation among waterbody samples and landscape variables are likely due to the recency of colonization, low number of occupied and sampled waterbodies (low replication), and low complexity of landscape variables quantified. Importantly, we have developed raster files for future analyses. We recommend that similar analyses continue when larger sample sizes of individuals and waterbodies are available.

*Procambarus clarkii* has been able to thrive across the globe in climates much different than in its native range (Peruzza et al. 2015; Veselý et al. 2015; Sato et al. 2023). A recent study identified genetic changes likely associated with higher cold tolerance in populations in northern Japan (Sato et al. 2023). It would be interesting to see if the same temporal genetic changes would occur in SE Michigan populations that similarly experience low temperatures and waterbodies that freeze in the winter. Understanding how climate change, including notable increases in temperature and changes in precipitation, affects populations in SE Michigan will also be imperative for effective management and control of the species going forward.

## Author Contributions

**Nicole E. Adams:** conceptualization (supporting), data curation (lead), formal analysis (equal), investigation (lead), methodology (equal), project administration (supporting), supervision (supporting), validation (equal), visualization (lead), writing – original draft (lead), writing – review and editing (equal). **Jared J. Homola:** conceptualization (lead), data curation (lead), formal analysis (equal), funding acquisition (supporting), investigation (lead), methodology (equal), project administration (supporting), supervision (supporting), validation (equal), visualization (equal), writing – original draft (equal), writing – review and editing (equal). **Nicholas M. Sard:** methodology (supporting), validation (equal), writing – review and editing (equal). **Lucas R. Nathan:** conceptualization (supporting), methodology (supporting), validation (equal), writing – original draft (supporting), writing – review and editing (equal). **Brian M. Roth:** conceptualization (supporting), methodology (supporting), validation (equal), writing – original draft (supporting), writing – review and editing (equal). **John D. Robinson:** conceptualization (lead), data curation (supporting), formal analysis (equal), funding acquisition (lead), methodology (equal), project administration (lead), supervision (lead), validation (equal), visualization (equal), writing – original draft (equal), writing – review and editing (equal). **Kim T. Scribner:** conceptualization (lead), data curation (supporting), formal analysis (supporting), funding acquisition (lead), methodology (equal), project administration (lead), supervision (lead), validation (equal), visualization (supporting), writing – original draft (equal), writing – review and editing (equal).

## Funding

This work was supported by the Michigan Invasive Species Grant Program, MISGP–PR5760.

## Conflicts of Interest

The authors declare no conflicts of interest.

## Supporting information


**Data S1:** ece372550‐sup‐0001‐AppendixS1.docx.

**Data S2:** ece372550‐sup‐0002‐AppendixS2.docx.

## Data Availability

Raw FASTQ files and barcodes for demultiplexing RAD data are available on Dryad (DOI: https://doi.org/10.5061/dryad.djh9w0wcq). Individual sequences after demultiplexing libraries are available on the NCBI sequence read archive (SRA) under bioproject PRJNA1148680. Code used for bioinformatics and data analysis is available at https://github.com/NicoleAdams‐sci/RedSwampCrayfish_geneflow.
