# Supplementary material for: Genomic Data Reveal Multiple Introduction Sources and Limited Post‐Colonization Gene Flow in Southeast Michigan Invasive Red Swamp Crayfish (Procambarus clarkii)
Source: Ecol Evol. 2025 Dec 9;15(12):e72550. doi: 10.1002/ece3.72550 (PMC12686971; doi:10.1002/ece3.72550)
Supplement: Supplementary file 1 — Data S1: ece372550‐sup‐0001‐AppendixS1.docx. [file ECE3-15-e72550-s001.docx]

**Genomic data reveal multiple introduction sources and limited post-colonization gene flow in southeast Michigan invasive Red Swamp Crayfish (*Procambarus clarkii*)**

Nicole E. Adams^1+*^, Jared J. Homola^1^, Nicholas M. Sard^3^, Lucas R. Nathan^4^, Brian M. Roth^1^, John D. Robinson^1^, Kim T. Scribner^1^

^1^ Department of Fisheries and Wildlife, Michigan State University, Natural Resources Building, 480 Wilson Rd., East Lansing, MI 48824, USA

^3^ Biological Sciences Department, The State University of New York – Oswego, 7060 State Route 104, Oswego, NY 13126, USA

^4^ Michigan Department of Natural Resources, 525 W. Allegan St, Lansing, MI 48933, USA

^+^Current address: Department of Ecology and Evolutionary Biology, University of Michigan, 105 N University Ave, Ann Arbor, MI 48109, USA

^*^Corresponding author: Nicole E. Adams (nicoleAdams.sci@gmail.com)

**Appendix S1**: Supporting methods

*DNA extraction and quantification*

Gill tissue was taken from each adult for DNA extraction. Genomic DNA was extracted from tissue using either a DNeasy Blood and Tissue kit (Qiagen, Hilden, Germany) following manufacturer protocols, or a bead-based protocol in 96-well plates following methods in Ali et al. (2016), see also Adams et al. (2024) for details. Initial DNA quantification was conducted using a spectrophotometer (NanoDrop 1000; Thermo-Fisher Scientific; Waltham, MA, USA) to ensure DNA concentrations were within the effective range for fluorometer measurements. DNA concentrations were subsequently measured with Quant-iT PicoGreen dsDNA Assay Kits (Invitrogen; Waltham, MA, USA) using a QuantStudio 6 Real-Time PCR System (Thermo-Fisher Scientific). DNA concentrations were standardized across samples to <80 ng/uL prior to RAD library construction.

*Library preparation and sequencing*

RAD capture libraries were prepared using the BestRAD protocol (Ali et. al., 2016) using *SbfI* restriction enzyme (New England Biolabs). BestRAD adapters (New England Biolabs, Ipswich, MA, USA) were ligated to the cut ends using T4 Ligase (New England Biolabs). Pooled samples were eluted in 135 µl of Low Tris-EDTA (TE) buffer and then sheared with a sonicator (Covaris M220, Woburn, MA, USA) for an average target fragment length of 325 base pairs (bp). Barcoded, sheared DNA was isolated with streptavidin beads (Dynabeads M-280, Invitrogen), then libraries were prepared following NEBNext Ultra (or Ultra II for plates 13-24) Library prep kit for Illumina (New England Biolabs). Libraries were dual-indexed using NEB Dual Index Sets 1-4 and amplified for 12 cycles, followed by a magnetic bead-based (Ampure XP bead, Beckman Coulter, Brea, CA, USA) cleanup. Libraries were quantified using a fluorometer (Qubit, Thermo Fisher Scientific), and the quality was assessed using microfluidic automated electrophoresis (Bioanalyzer, Agilent, Santa Clara, CA, USA).

Capture reactions were carried out using the MyBaits Version 4.01(Daicel Arbor Biosciences; Ann Arbor, MI, USA). For more information on the development and use of the RAD capture baits see Adams et al. (2024).

Capture reactions were amplified for 11 cycles using KAPA Library Amplification Kit for Illumina (KAPA Biosystems, Wilmington, MA, USA) and quantified using a Qubit. Quality was assessed using automated electrophoresis (TapeStation; Aligent). The four capture libraries were then pooled into one sequencing library that included equal DNA quantities from each of the capture libraries, which was then assessed with Qubit and a TapeStation. The RAD capture library was then sequenced on one lane of Illumina's NovaSeq 6000 as 150 bp paired-end reads at the Research Technology Support Facility (RTSF) Genomics Core at MSU. We created 36 total libraries for two independent sequencing runs. The first 12 were sequenced in 2021 and the remaining 24 were sequenced in 2022. These libraries included additional samples of *P. clarkii* for a previous project (Adams et al. 2024) that were simultaneously prepared and sequenced. Individual sequences after demultiplexing libraries are available on the NCBI Sequence Read Archive (Bioproject PRJNA1148680).

*Filtering variant data*

To remove the most poorly sequenced samples and loci, we first used VCFtools to filter out SNPs with quality scores less than a minimum of 20, minor allele counts less than three, greater than 99% missing data, and read counts of two or fewer. We then removed individuals with more than 99% missing data. Next, we used an iterative filtering approach in which we increased stringency in the maximum missingness allowed across loci and individuals. We conducted 20 rounds of filtering starting with removing loci with more than 96% missing data and individuals with greater than 97.5% missing data. We increased the missingness stringency by 2.5% and 3.75% for loci and individuals, respectively, each filtering round. As the missingness stringency for individuals increased, the number of individuals removed from the dataset plateaued around the twelfth round of filtering so we chose those cutoffs: removed loci with greater than 55% missing data and removed individuals with more than 70% missing data (Fig. S1). For all iterative filtering rounds, we kept loci with a minimum depth coverage of 7x and a minor allele count greater than or equal to three. Then we removed loci with observed heterozygosity greater than 0.6 and allele balance values greater than 0.6 and less than 0.4 (McKinney et al., 2017). To minimize non-independence between loci, we kept only one SNP per RAD tag based on the least amount of missing data. If two or more SNPs met all the criteria in a RAD tag, the first SNP was selected. Since RAD tags could still be physically close to one another, we removed loci separated by less than 100 kb based on the reference genome assembly (Xu et al., 2021). We evaluated the transitions and transversions in the 2,675 SNPs used in the analyses of genetic diversity and gene flow dataset and found a transition/transversion (Ti/Tv) ratio of 1.533. This is lower than the ratio found in *P. clarkii* in a transcriptomics study which found a ratio of 1.96 (Shen et al., 2014). The differences could be attributed to many things, one of which is how each dataset was filtered and how much of the whole genome each represents.

***Demographic modeling***

We reconstructed the species’ invasion history in SE Michigan using a demographic modeling approach and the Approximate Bayesian Computation (ABC) statistical framework (Bertorelle et al., 2010; Csilléry et al., 2010). We compared the relative support for a series of competing models describing the invasion of *P. clarkii* in SE Michigan and estimated posterior distributions for demographic parameters, including effective population size and bottleneck severity, from the best-fit model. Leave-one-out cross validations were used to assess the level of confidence in results from the model selection and parameter estimation analyses.

*Model development*

We defined ten competing demographic models that conceptualized different hypotheses of the founding and secondary geographic spread of *P. clarkii* in southeastern Michigan (Fig. 2, Fig. S3). Using these models, we tested two general dispersal hypotheses: stepping-stone and bridgehead models. The one-dimensional stepping-stone model posits that dispersal and colonization occurred only between adjacent geographic regions (Kimura and Weiss, 1964). Alternatively, it has been shown empirically that in some species an initial colonized site can become the source of multiple secondary introductions (Grapputo et al., 2005; Darling et al., 2008; Ficetola et al., 2008). This pattern has been referred to as the “invasive bridgehead effect” (Lombaert et al., 2010; Bertelsmeier and Keller, 2018; Blumenfeld et al., 2021).

We evaluated support for stepping-stone and bridgehead scenarios focusing on Group 2 and Group 3 as potential secondary sources. Initial reports of *P. clarkii* in southeastern Michigan were from Group 2 (Smith et al., 2018), evidence for it being a potential source for secondary spread. Further, Group 2 and Group 3 have large numbers of *P. clarkii* individuals, and have a number of smaller populations in close proximity, which could also indicate that the SE Michigan colonization started in these groups. Based on the results of our ordination, unsupervised clustering, and neighbor-joining tree analyses, we modeled Groups 2 and 3 as recently diverged populations each with ongoing gene flow within both groups. Additionally, we tested a separate stepping-stone model based on a west to east dispersal pattern initiated from Group 1, based on its geographic location. The opposite hypothesis, an east to west stepping-stone model was already incorporated in the stepping-stone model with dispersal initiated from colonization from Group 3 and non-independent introductions of Groups 1 and 5. Our population genetic structure analyses also suggested that Group 1 and Group 5 were genetically distinct and potentially the most diverged from the other SE Michigan groups. Therefore, our models compared whether Groups 1 and 5 were part of the secondary dispersal event or whether these groups of sampled locations were separate introductions into the region. Our final model investigated whether there was support for five independent colonization events for Groups 1, 2, 3, 4, and 5. All of our models included an unsampled population in the native range that served as the source for the invasion of Michigan, reflecting the potential that the sampled waterbodies in SE Michigan may not have been colonized from the same native population sampled for this study. Raw DNA sequences for Louisiana samples were provided by Sard et al. (2023) and bioinformatically processed as above.

Our models assumed a one-year generation time for *P. clarkii* (Huner and Barr, 1991), a base pair (bp) mutation rate of 3.6 x 10^-9^ (Liu et al., 2016), and a 150 bp sequence length. We allowed high migration between the unsampled source population in the native range and our sampled population from Louisiana. Within a group with multiple genetic clusters, we also allowed migration, but at a lower rate than in the native range based on our clustering results and movement data (Adams et al., 2024).

*Simulations and statistical analyses*

We compared the alternative demographic models described above using ABC and coalescent simulations. Coalescent simulations for each of the ten competing models were conducted in fastsimcoal2 v 2.709 (Excoffier et al., 2021) using modified wrapper functions from the strataG package (v 2.5.5 Archer et al., 2017) in R v 4.1.2 (R Core Team, 2016). Functions available in strataG and custom scripts were used to calculate summary statistics for each simulated replicate. Competing models were simulated 100,000 times each for ABC‐based model selection and parameter estimation (Bertorelle et al., 2010). Simulations under each model used parameter values randomly drawn from prior distributions (Table S3). Model parameters included local effective population sizes in each group, migration rates between populations within regions (i.e., within Group 2 and 3), and measures of the severity of population bottlenecks associated with colonization (see Table S3 for a full list). Our ABC analyses used comparisons between summary statistics calculated from the observed dataset and those derived from simulated datasets to evaluate support for alternative models and estimate parameter values for supported models. Due to the missing data in the empirical dataset for demographic modeling described above, we removed genotype calls in simulated datasets to match the per-locus and per-population pattern of missing data in our observed *P. clarkii* genotypic dataset. We calculated metrics of intra-population diversity including: the number of polymorphic loci (S) in each population, a measure of allelic richness based on the number of observed alleles in the population and the number of genotyped samples (rS) per population (strataG; Archer et al., 2017), the number of private polymorphic loci (pS; strataG; Archer et al., 2017), the frequency-down-weighted loci value (Schönswetter and Tribsch, 2005) per population, and the expected heterozygosity (He) in each population. Additionally, we calculated metrics of inter-population diversity and measures of population divergence including pairwise private loci (i.e., those falling along the axes of the joint site frequency spectrum for each pair of populations; Sard et al. 2019) and pairwise F_ST_ (Nei, 1987). A linear model was fitted between transformed F_ST_ and the natural log of geographic distance (Rousset, 1997) to test for patterns of isolation-by-distance (Wright, 1943). To evaluate the direction of colonization during the Michigan *P. clarkii* invasion, we calculated the directionality index (Ѱ; Peter and Slatkin, 2013), which compares minor allele frequencies at shared polymorphic loci between pairs of populations. In theory, populations that serve as the source of introduced individuals are expected to have lower minor allele frequencies at these loci (Peter and Slatkin, 2013; 2015).

*Evaluating model fit and estimating parameters*

Approximate Bayesian Computation analyses were conducted using the R packages abc (Csilléry, François, & Blum, 2012) and abcrf (Marin, Raynal, Pudlo, Robert, & Estoup, 2017). Model selection analyses were done using multinomial logistic regression, neural network (Blum & Francois, 2010), and random forest (Pudlo et al., 2015) methods. Selection analyses using multinomial logistic regression and neural network methods were conducted with three tolerance values: 0.005 (5,000 accepted simulations), 0.01 (10,000 accepted simulations), 0.025 (25,000 accepted simulations). Ten neural networks were used along with 20 hidden layers in the neural network analysis. The random forest analysis used 1,000 trees to predict the most likely model.

To evaluate model selection outcomes under ABC, one hundred leave-one-out cross‐validation replicates were conducted for each of the simulated models using multinomial logistic regression and neural networks with tolerances of 0.005 and 0.025, respectively. Out-of-bag (OOB) error rates were calculated to assess performance of the random forest ABC model selection analysis.

Parameter values were estimated using the most supported models. Specifically, for each group (with Groups 2 and 3 split into their two genetic clusters), we estimated effective population sizes, a bottleneck severity value, and migration rates within Michigan using a neural network approach and 0.05 and 0.1 tolerances (5000, 10,000 simulations). Leave-one-out cross-validations were used to characterize confidence in parameter estimates from ABC analyses. We conducted 10 leave-one-out cross-validation replicates for the best supported models using a neural network method with a tolerance of 0.05.

**REFERENCES**

Adams, N. E., Homola, J. J., Sard, N. M., Nathan, L. R., Roth, B. M., Robinson, J. D., & Scribner, K. T. (2024). Genomic data characterize reproductive ecology patterns in Michigan Invasive Red Swamp Crayfish (*Procambarus clarkii*). *Evolutionary Applications*, *17*(9). https://doi.org/10.1111/EVA.70007

Ali, O. A., O’Rourke, S. M., Amish, S. J., Meek, M. H., Luikart, G., Jeffres, C., & Miller, M. R. (2016). RAD capture (Rapture): flexible and efficient sequence-based genotyping. *Genetics*, 202(2), 389-400.

Archer, F. I., Adams, P. E., & Schneider, B. B. (2017). STRATAG: An R package for manipulating, summarizing and analysing population genetic data. *Molecular Ecology Resources*, *17*, 5–11. doi: 10.1111/1755-0998.12559

Bertelsmeier, C., & Keller, L. (2018). Bridgehead effects and role of adaptive evolution in invasive populations. *Trends in Ecology & Evolution*, *33*(7). doi: 10.1016/j.tree.2018.04.014

Bertorelle, G., Benazzo, A., & Mona, S. (2010). ABC as a flexible framework to estimate demography over space and time: some cons, many pros. *Molecular Ecology*, *19*, 2609–2625. doi: 10.1111/j.1365-294X.2010.04690.x

Blum, M. G. B., & François, O. (2010). Non-linear regression models for Approximate Bayesian Computation. *Statistics and Computing*, *20*(1), 63–73. doi: 10.1007/s11222-009-9116-0

Blumenfeld, A. J., Eyer, P. A., Husseneder, C., Mo, J., Johnson, L. N. L., Wang, C., … Vargo, E. L. (2021). Bridgehead effect and multiple introductions shape the global invasion history of a termite. *Communications Biology*, *4*(1), 1–12. doi: 10.1038/s42003-021-01725-x

Csilléry, K., Blum, M. G. B., Gaggiotti, O. E., & François, O. (2010). Approximate Bayesian Computation (ABC) in practice. *Trends in Ecology and Evolution*, *25*(7), 410–418. doi: 10.1016/j.tree.2010.04.001

Csilléry, K., François, O., & Blum, M. G. B. (2012). abc: an R package for approximate Bayesian computation (ABC). *Methods in Ecology and Evolution*, *3*, 475–479. doi: 10.1111/j.2041-210X.2011.00179.x

Darling, J. A., Bagley, M. J., Roman, J., Tepolt, C. K., & Geller, J. B. (2008). Genetic patterns across multiple introductions of the globally invasive crab genus *Carcinus*. *Molecular Ecology*, *17*, 4992–5007. doi: 10.1111/j.1365-294X.2008.03978.x

Excoffier, L., Marchi, N., Marques, D. A., Matthey-Doret, R., Gouy, A., & Sousa, V. C. (2021). fastsimcoal2: demographic inference under complex evolutionary scenarios. *Bioinformatics*, *37*(24), 4882–4885. doi: 10.1093/bioinformatics/btab468

Ficetola, G. F., Bonini, A., & Miaud, C. (2008). Population genetics reveals origin and number of founders in a biological invasion. *Molecular Ecology*, *17*, 773–782. doi: 10.1111/j.1365-294X.2007.03622.x

Grapputo, A., Boman, S., Lindström, L., Lyytinen, A., & Mappes, J. (2005). The voyage of an invasive species across continents: genetic diversity of North American and European Colorado potato beetle populations. *Molecular Ecology*, *14*, 4207–4219. doi: 10.1111/j.1365-294X.2005.02740.x

Huner, J. V., & Barr, J. E. (1991). Red Swamp Crayfish: Biology and Exploitation. 3rd edition. Louisiana Sea Grant College Program.

Kimura, M., & Weisss, G. H. (1964). The stepping stone model of population structure and decrease of genetic correlations with distance. *Genetics*, *49*, 561–576. doi: 10.1093/genetics/49.4.561

Liu, H., Jia, Y., Sun, X., Tian, D., Hurst, L. D., & Yang, S. (2016). Direct determination of the mutation rate in the bumblebee reveals evidence for weak recombination-associated mutation and an approximate rate constancy in insects. *Molecular Biology and Evolution*, *34*(1), 119–130. doi: 10.1093/molbev/msw226

Lombaert, E., Guillemaud, T., Cornuet, J.-M., Malausa, T., Facon, B. B., & Estoup, A. (2010). Bridgehead effect in the worldwide invasion of the biocontrol harlequin ladybird. *Plos One*, *5*(3), e9743. doi: 10.1371/journal.pone.0009743

Marin, J. M., Raynal, L., Pudlo, P., Robert, C. P., & Estoup, A. (2017). *abcrf: Approximate Bayesian Computation via Random Forests.* Retrieved from https://cran.r‐project.org/ package=abcrf

McKinney, G. J., Waples, R. K., Seeb, L. W., & Seeb, J. E. (2017). Paralogs are revealed by proportion of heterozygotes and deviations in read ratios in genotyping-by-sequencing data from natural populations. *Molecular Ecology Resources*, *17*(4), 656–669. doi: 10.1111/1755-0998.12613

Nei, M. (1987). *Molecular Evolutionary Genetics*. Columbia University Press.

Peter, B. M., & Slatkin, M. (2013). Detecting range expansions from genetic data. *Evolution*, *67*(11), 3274–3289. doi: 10.1111/evo.12202

Peter, B. M., & Slatkin, M. (2015). The effective founder effect in a spatially expanding population. *Evolution*, 69(3), 721–734. doi: 10.1111/EVO.12609

Pudlo, P., Marin, J.-M., Estoup, A., Cornuet, J.-M., Gautier, M., & Robert, C. P. (2016). Reliable ABC model choice via random forests. *Bioinformatics*, *32*(6), 859–866. doi: 10.1093/bioinformatics/btv684

Rousset, F. (1997). Genetic differentiation and estimation of gene flow from F-statistics under isolation by distance. *Genetics*, *145*, 1219–1228.

Sard, N., Robinson, J., Kanefsky, J., Herbst, S., & Scribner, K. (2019). Coalescent models characterize sources and demographic history of recent round goby colonization of Great Lakes and inland waters. *Evolutionary Applications*, *12*(5), 1034–1049. doi: 10.1111/eva.12779

Sard, N. M., Smith, K. R., Roth, B. M., Nathan, L. R., Herbst, S. J., & Scribner, K. T. (2023). Multiple sources implicated in the red swamp crayfish invasion in Michigan, USA. *Biological Invasions*, (0123456789). doi: 10.1007/s10530-022-02938-9

Schönswetter, P., & Tribsch, A. (2005). Vicariance and dispersal in the alpine perennial *Bupleurum stellatum* L. (Apiaceae). *Taxon*, *54*(3), 725–732. https://doi.org/10.2307/25065429

Shen, H., Hu, Y., Ma, Y., Zhou, X., Xu, Z., Shui, Y., Li, C., Xu, P., & Sun, X. (2014). In-Depth Transcriptome Analysis of the Red Swamp Crayfish *Procambarus clarkii*. *PLOS ONE*, 9(10), e110548. https://doi.org/10.1371/journal.pone.0110548

Smith, K., Roth, B. M., Herbst, S. J., Thoma, R. F., Popoff, N., Hayes, D. B., & Jones, M. L. (2018). Assessment of invasion risks for Red Swamp Crayfish (*Procambarus clarkii*) in Michigan, USA. *Management of Biological Invasions*, *9*, 405–415. doi: 10.3391/mbi.2018.9.4.04

Wright, S. (1943). Isolation-by-distance. *Genetics*, *28*(2), 114–138.

Xu Z, Gao T, Xu Y, Li X, Li J, Lin H, Yan W, Pan J, Tang J. A chromosome-level reference genome of Red Swamp Crayfish *Procambarus clarkii* provides insights into the gene families regarding growth or development in crustaceans. *Genomics*. 2021 Sep;113(5):3274-3284. doi: 10.1016/j.ygeno.2021.07.017
