# Supplementary material for: Genomic Data Reveal Multiple Introduction Sources and Limited Post‐Colonization Gene Flow in Southeast Michigan Invasive Red Swamp Crayfish (Procambarus clarkii)
Source: Ecol Evol. 2025 Dec 9;15(12):e72550. doi: 10.1002/ece3.72550 (PMC12686971; doi:10.1002/ece3.72550)
Supplement: Supplementary file 2 — Data S2: ece372550‐sup‐0002‐AppendixS2.docx. [file ECE3-15-e72550-s002.docx]

**SUPPLEMENTARY**

*Supplementary tables and figures*

Table S1. Summary of the number of sequenced Red Swamp Crayfish samples by waterbody and year sampled. The samples from Louisiana in the species’ native range were sequenced for an earlier project (see Sard et al., 2023).

| **Group** | **Site** | **Abbreviation** | 2021 | 2022 | **Total samples** |
| --- | --- | --- | --- | --- | --- |
| 1 | Apartment 1 | Apt1 | 25 | 19 | 44 |
|  | Apartment 2 | Apt2 | 2 | 21 | 23 |
|  | Apartment 3 | Apt3 | 4 | 8 | 12 |
| 2 | West Golf Course 1 | WestGC1 | 50 | 106 | 156 |
|  | West Golf Course 2 | WestGC2 | 30 | 29 | 59 |
|  | West Golf Course 3 | WestGC3 | 1 | 4 | 5 |
|  | West Golf Course 6 | WestGC6 | 21 | 10 | 31 |
|  | Hotel 1 | Hotel1 | 53 | 80 | 133 |
|  | Hotel 2 | Hotel2 | 11 | — | 11 |
|  | Hotel 3 | Hotel3 | 30 | 11 | 41 |
|  | Hotel 4 | Hotel4 | 29 | 13 | 42 |
|  | Hotel 5 | Hotel5 | 1 | 1 | 2 |
| 3 | East Golf Course 1 | EastGC1 | 52 | 6 | 58 |
|  | East Golf Course 2 | EastGC2 | 50 | 54 | 104 |
|  | East Golf Course 3 | EastGC3 | 47 | 10 | 57 |
|  | East Golf Course 4 | EastGC4 | 57 | 63 | 120 |
|  | East Golf Course 7 | EastGC7 | 43 | 20 | 63 |
|  | East Golf Course 8 | EastGC8 | — | 1 | 1 |
|  | East Golf Course 9 | EastGC9 | — | 1 | 1 |
|  | East Golf Course 10 | EastGC10 | 30 | 30 | 60 |
|  | East Golf Course 11 | EastGC11 | 30 | 55 | 85 |
|  | East Golf Course 13 | EastGC13 | — | 48 | 48 |
| 4 | Private 1 | Private1 | 30 | 8 | 38 |
|  | Private 2 | Private2 | 1 | 1 | 2 |
| 5 | Drain 1 | Drain1 | 36 | 28 | 64 |
|  | Louisiana* | LOA | — | — | 17 |
|  | ***P. clarkii* total by year** |  | 454 | 627 | 1277 |
|  | *Creaserinus fodiens* | Cfod | — | 3 | 3 |
|  | *Cambarus thomai (Lacunicambarus thomai)* | Ctho | — | 3 | 3 |
|  | *Faxonius propinquus* | Fprop | — | 1 | 1 |
|  | *Faxonius virilis* | Fvir | — | 1 | 1 |
|  | *Lacunicambarus polychromatus* | Lpol | — | 1 | 1 |
|  | *Procambarus acutus* | Pacu | — | 1 | 1 |
|  | **Total** |  |  | 637 | 1287 |

Table S2. Filtering steps used to create the SNP data set for analyses of genetic diversity and gene flow as well as the dataset used in the ABC demographic analysis. The program(s) and main parameter(s) used for each filtering step are shown with the resulting number of individuals and number of SNPs.

|  |  |  | Gene flow | | ABC | |
| --- | --- | --- | --- | --- | --- | --- |
| Step | Program | Parameters | Individuals | SNPs | Individuals | SNPs |
| Create VCF | Stacks | populations | 1,255 | 521,238 | 1284 | 967,333 |
| Remove non-P. Clarkii samples | VCFtools | --remove otherSP.txt | — | — | 1278 | 967,333 |
| Basic filters | VCFtools | --minGQ 20 --max-missing 0.01 --min-meanDP 2 | 1,255 | 102,491 | 1278 | 155,655 |
| Remove individuals with high missingness | VCFtools | --missing-indv (< 0.99) | 1,249 | 102,491 | 1275 | 155,655 |
| Iterative filtering | VCFtools | --minDP 7 --minGQ 20 --mac 3 --max-missing 0.45, --missing-indv (< 0.70) | 763 | 27,054 | — | — |
| Filter based on missingness | VCFtools | --minDP 5 --max-missing 0.50, --missing-indv (< 0.75) | — | — | 948 | 47,149 |
| Filter excess heterozygosity | VCFtools/R | Ho <= 0.6 | 763 | 26,778 | 948 | 22,171 |
| Filter on allele balance | R/VCFtools | AB > 0.4, AB < 0.6 | 763 | 21,859 | 948 | 13,523 |
| Filter 1 SNP per RAD tag | R/VCFtools | — | 763 | 3,497 | 948 | 2,212 |
| Filter SNPs within 100kb distance per chromosome | R/VCFtools | — | 763 | 2,675 | 948 | 1,808 |
| Filter populations with N<5 (only for population-level analyses) | R | — | 757 | 2,675 | — | — |
| Remove loci and individuals > 20% missing | R (adegenet, poppr) | — | 653 | 1,315 | — | — |

Table S3. Modeled parameters and their definitions and minimum and maximum prior values used in demographic modeling.

| Parameter | Definition | Prior distribution |
| --- | --- | --- |
| G | Generation time | 1 |
| mu | Mutation rate | 3.6e-9 |
| N.LA | Effective population size of the sampled Louisiana population | 100, 10000* |
| N.UNK | Effective population size of the unsampled native range population | 100, 10000* |
| N.Grp1 | Effective population size of Group 1 | 10, 2000* |
| N.Grp2_WestGC | Effective population size of one of the genetic groups in Group 2 | 10, 2000* |
| N.Grp2_Hotel | Effective population size of the other genetic group in Group 2 | 10, 2000* |
| N.Grp3_NE | Effective population size of the northeast genetic group in Group 3 | 10, 2000* |
| N.Grp3_SW | Effective population size of the southwest genetic group in Group 3 | 10,2000* |
| N.Grp4 | Effective population size of Group 4 | 10, 2000* |
| N.Grp5 | Effective population size of Group 5 | 10, 2000* |
| m.NA | Migration rate between the sampled Louisiana and unsampled native range | 0.01, 0.05^♢^ |
| m.Grp2 | Migration rate within Group 2 | 0.0001, 0.01* |
| m.Grp3 | Migration rate within Group 3 | 0.0001, 0.01* |
| T.UNK | Time of coalescence to LA pop | 1000, 5000^♢^ |
| T.MI | Time of southeastern Michigan population | 50 |
| bott.sev | Bottleneck strength | 0.001, 0.25* |

* log uniform distribution, ^♢^ uniform distribution

Table S4. Sex-specific measures of genetic diversity for invasive *P. clarkii* populations in SE Michigan. For each waterbody, genetic diversity metrics were calculated separately for males and females: allelic richness (Ar), number of private alleles (Ap), mean observed heterozygosity (Ho), mean expected heterozygosity (He), and inbreeding coefficient (F_IS_). Sample sizes indicate the number of females (F) and males (M) used in each analysis. Dashes (—) indicate insufficient sample size for reliable estimation (N<3 for Ar and FIS, N<5 for Ap). Groups are based on levels of genetic differentiation and geographic proximity (see Fig. 1). Full names of waterbodies can be found in Table S1.

| Group | Waterbody | N (F,M) | A_r_ | | A_p_ | | H_o_ | | H_e_ | | F_IS_ | |
| --- | --- | --- | --- | --- | --- | --- | --- | --- | --- | --- | --- | --- |
|  |  |  | Female | Male | Female | Male | Female | Male | Female | Male | Female | Male |
| 1 | Apt1 | 24 (10, 14) | 1.244 | 1.247 | 159 | 52 | 0.081 | 0.082 | 0.089 | 0.088 | 0.0743 | 0.0656 |
|  | Apt 2 | 6 (2,4) | — | *—* | 0 | 0 | 0.06 | 0.062 | 0.051 | 0.069 | — | 0.0612 |
|  | Apt 3 | 6 (1,5) | *—* | *—* | 0 | 4 | 0.075 | 0.07 | — | 0.077 | — | 0.0506 |
| 2 | WestGC1 | 69 (36,33) | 1.098 | 1.1 | 667 | 607 | 0.028 | 0.031 | 0.031 | 0.032 | 0.0605 | 0.0403 |
|  | WestGC2 | 33 (15,18) | 1.103 | 1.1 | 1 | 0 | 0.029 | 0.029 | 0.033 | 0.032 | 0.0675 | 0.0395 |
|  | WestGC6 | 18 (7,11) | 1.147 | 1.122 | 0 | 2 | 0.036 | 0.036 | 0.039 | 0.039 | 0.0372 | 0.0452 |
|  | Hotel1 | 108 (49,59) | 1.045 | 1.046 | 0 | 1 | 0.016 | 0.017 | 0.018 | 0.018 | 0.0938 | 0.0664 |
|  | Hotel2 | 11 (4,7) | *—* | 1.049 | 1 | 1 | 0.016 | 0.016 | 0.016 | 0.016 | -0.0327 | 0.0172 |
|  | Hotel3 | 27 (16,11) | 1.049 | 1.046 | 0 | 0 | 0.016 | 0.016 | 0.018 | 0.018 | 0.0715 | 0.1025 |
|  | Hotel4 | 30 (10,20) | 1.044 | 1.045 | 0 | 0 | 0.016 | 0.016 | 0.016 | 0.017 | 0.008 | 0.0372 |
| 3 | EastGC1 | 8 (6,2) | 1.213 | *—* | 1 | 0 | 0.032 | 0.03 | 0.034 | 0.031 | 0.0291 | — |
|  | EastGC2 | 69 (43,26) | 1.105 | 1.107 | 0 | 1 | 0.03 | 0.027 | 0.032 | 0.03 | 0.0531 | 0.0642 |
|  | EastGC3 | 9 (5,4) | 1.088 | 1.074 | 0 | 2 | 0.024 | 0.021 | 0.027 | 0.024 | 0.112 | 0.0859 |
|  | EastGC4 | 51 (27,24) | 1.083 | 1.088 | 0 | 0 | 0.027 | 0.025 | 0.027 | 0.029 | 0.0191 | 0.1224 |
|  | EastGC7 | 28 (20,8) | 1.101 | 1.092 | 0 | 0 | 0.03 | 0.031 | 0.032 | 0.031 | 0.0399 | 0.003 |
|  | EastGC10 | 33 (14,19) | 1.085 | 1.083 | 0 | 0 | 0.027 | 0.025 | 0.028 | 0.028 | 0.0463 | 0.0875 |
|  | EastGC11 | 27 (14,13) | *—* | *—* | 29 | 32 | 0.028 | 0.03 | 0.031 | 0.029 | 0.0474 | -0.0598 |
|  | EastGC13 | 35 (21,14) | 1.084 | 1.086 | 1 | 3 | 0.027 | 0.027 | 0.028 | 0.028 | 0.0427 | 0.0405 |
| 4 | Private1 | 25 (8,17) | 1.072 | 1.068 | 1 | 0 | 0.03 | 0.028 | 0.028 | 0.028 | -0.0852 | 0.0006 |
| 5 | Drain1 | 36 (22,14) | 1.534 | 1.528 | 1 | 3 | 0.148 | 0.146 | 0.162 | 0.16 | 0.0668 | 0.0596 |

Table S5. Results from analyses evaluating evidence for significant inbreeding (F_IS_; deviation from observed and expected heterozygosity) within each waterbody, using a t-test. Significant results are in bold text. Groups are based on genetic differentiation and geographic proximity (see Fig. 1). Full names of waterbodies can be found in Table S1.

| Group | Waterbody | N | F_IS_ | t | DF | P-value |
| --- | --- | --- | --- | --- | --- | --- |
| 1 | Apt1 | 24 | 0.079 | 6.055 | 378 | **3.39 x 10^-9^** |
|  | Apt3 | 6 | 0.038 | 1.63 | 269 | 0.1051 |
|  | Apt2 | 6 | 0.095 | 3.466 | 217 | **6.36 x 10^-4^** |
| 2 | WestGC1 | 69 | 0.052 | 4.181 | 200 | **4.33 x 10^-5^** |
|  | WestGC2 | 33 | 0.056 | 3.828 | 193 | **1.75 x 10^-4^** |
|  | WestGC6 | 18 | 0.049 | 3.018 | 205 | **2.871 x 10^-3^** |
|  | Hotel1 | 108 | 0.076 | 3.717 | 81 | **3.7 x 10^-4^** |
|  | Hotel2 | 11 | 0.003 | 0.084 | 58 | 0.933 |
|  | Hotel3 | 27 | 0.08 | 3.317 | 78 | **1.383 x 10^-3^** |
|  | Hotel4 | 30 | 0.029 | 1.191 | 76 | 0.237 |
| 3 | EastGC1 | 8 | 0.042 | 1.601 | 138 | 0.112 |
|  | EastGC7 | 28 | 0.058 | 3.688 | 199 | **2.92 x 10^-4^** |
|  | EastGC10 | 33 | 0.1 | 5.252 | 162 | **4.67 x 10^-7^** |
|  | EastGC11 | 27 | 0.073 | 3.607 | 156 | **4.17 x 10^-4^** |
|  | EastGC13 | 35 | 0.033 | 2.084 | 198 | **0.038** |
|  | EastGC2 | 69 | 0.064 | 4.561 | 167 | **9.83 x 10^-6^** |
|  | EastGC3 | 9 | 0.027 | 0.896 | 130 | 0.3721 |
|  | EastGC4 | 51 | 0.042 | 2.877 | 162 | **4.56** **x 10^-3^** |
| 4 | Private1 | 25 | -0.017 | -0.816 | 99 | 0.417 |
| 5 | Drain1 | 36 | 0.07 | 10.002 | 950 | **< 2.2 x 10^-16^** |

N=sample size, t=t-test statistic, DF=degrees of freedom

Table S6. Landscape genetics model selection results after a bootstrap analysis using 1000 iterations. The models are sorted by average AICc values (lowest first). Here we show the number of parameters (k), the difference in the avg AICc values (ΔAICc) between the best supported model (lowest AICc) and each subsequent model, the average of the AICc weights from 1,000 bootstrap iterations, and the average marginal R^2^ of 1,000 bootstrap iterations for each model.

|  | Predictor(s) | Avg AICc | k | ΔAICc | Avg weight | Avg marginal R^2^ |
| --- | --- | --- | --- | --- | --- | --- |
| Group 2 | Distance | -29.0830 | 2 | — | 1 | 0.7000 |
|  | Hydrography | 2.5301 | 3 | 31.6 | 0 | 0.2151 |
|  | Canopy | 9.0729 | 4 | 38.2 | 0 | 0.7051 |
|  | Roads | 28.5501 | 5 | 57.6 | 0 | 0.7993 |
|  | Canopy + Hydrography | 56.2597 | 6 | 85.3 | 0 | 0.6944 |
|  | Roads + Hydrography | 84.5646 | 7 | 113.6 | 0 | 0.8024 |
|  | Canopy + Roads | 118.1342 | 8 | 147.2 | 0 | 0.8124 |
|  | NLCD | 124.1894 | 8 | 153.3 | 0 | 0.5897 |
|  | Canopy + Roads + Hydrography | 198.8764 | 10 | 228.0 | 0 | 0.7937 |
|  | Canopy + Hydrography + NLCD + Roads | 601.9383 | 17 | 631.0 | 0 | 0.8668 |
| Group 3 | Distance | -52.9564 | 2 | — | 0.8741 | 0.2996 |
|  | Roads | -45.3205 | 3 | 7.6 | 0.0539 | 0.4128 |
|  | Hydrography | -41.9574 | 3 | 11 | 0.0719 | 0.3048 |
|  | Canopy | -12.8574 | 4 | 40.1 | 0 | 0.3151 |
|  | Roads + Hydrography | 7.9217 | 5 | 60.9 | 0 | 0.3847 |
|  | NLCD | 8.9816 | 5 | 61.9 | 0 | 0.3086 |
|  | Canopy + Hydrography | 34.0494 | 6 | 87 | 0 | 0.3805 |
|  | Canopy + Roads | 35.1728 | 6 | 88.1 | 0 | 0.3113 |
|  | NLCD + Roads | 62.9556 | 7 | 115.9 | 0 | 0.3911 |
|  | NLCD + Hydrography | 63.56 | 7 | 116.5 | 0 | 0.3922 |
|  | Canopy + Roads + Hydrography | 98.0347 | 8 | 151 | 0 | 0.3836 |
|  | NLCD + Canopy | 99.3569 | 8 | 152.3 | 0 | 0.3074 |
|  | Roads + Hydrography + NLCD | 135.8708 | 9 | 188.8 | 0 | 0.3834 |
|  | Canopy + Hydrography + NLCD | 178.6691 | 10 | 231.6 | 0 | 0.3715 |
|  | Canopy + Roads + NLCD | 179.4288 | 10 | 232.4 | 0 | 0.3058 |
|  | Canopy + Hydrography + NLCD + Roads | 273.7766 | 12 | 326.7 | 0 | 0.3909 |

Table S7. Random forest confusion matrix showing the number of times our ten models were assigned correctly (diagonal) or incorrectly. Class error is the out-of-bag error, or how often a model was misclassified. Models with secondary spread starting from Group 2 are indicated with “Grp2” and from Group 3 as “Grp3”. “Step” indicates the models based on stepping-stone versus bridgehead (“bridge”) models. Models that had an independent Group 1 and Group 5 introduction are indicated with “indGrp1_5”. The model with independent colonizations for Groups 1, 2, 3, 4, and 5 is labeled “5cols”. The model of secondary spread from west to east is labeled as “W2E”. For additional model details see Fig. S3.

|  |  | Estimated | | | | | | | | | |  |
| --- | --- | --- | --- | --- | --- | --- | --- | --- | --- | --- | --- | --- |
|  |  | 5cols_step | Grp3_bridge | Grp3_bridge_indGrp1_5 | Grp3_step | Grp3_step__indGrp1_5 | Grp2_bridge | Grp2_bridge_indGrp1_5 | Grp2_step | Grp2_step_indGrp1_5 | W2E_step | Class error |
| Simulated | 5cols_step | 92160 | 1209 | 2254 | 127 | 280 | 1109 | 2285 | 107 | 317 | 152 | 0.078 |
|  | Grp3_bridge | 2045 | 74948 | 2822 | 4755 | 618 | 4925 | 170 | 4817 | 458 | 4442 | 0.251 |
|  | Grp3_bridge_indGrp1_5 | 5872 | 1360 | 67462 | 108 | 11728 | 180 | 4515 | 33 | 7810 | 932 | 0.325 |
|  | Grp3_step | 1175 | 7343 | 133 | 82235 | 75 | 3294 | 80 | 4607 | 30 | 1028 | 0.178 |
|  | Grp3_step_indGrp1_5 | 2610 | 474 | 10838 | 189 | 75477 | 192 | 5433 | 29 | 3854 | 904 | 0.245 |
|  | Grp2_bridge | 1899 | 5323 | 218 | 4919 | 490 | 71348 | 2400 | 4993 | 624 | 7786 | 0.287 |
|  | Grp2_bridge_indGrp1_5 | 5380 | 151 | 4783 | 47 | 8139 | 1333 | 65553 | 124 | 11843 | 2647 | 0.344 |
|  | Grp2_step | 1216 | 4008 | 107 | 4475 | 25 | 6686 | 102 | 82431 | 79 | 871 | 0.176 |
|  | Grp2_step_indGrp1_5 | 2645 | 211 | 6181 | 27 | 3594 | 490 | 10166 | 183 | 75168 | 1335 | 0.248 |
|  | W2E_step | 1523 | 2152 | 653 | 1285 | 1598 | 5371 | 4139 | 1091 | 1375 | 80813 | 0.192 |

Table S8. Weighted median estimates for 10 focal parameters for our four most supported demographic models: stepping-stone (step) and bridgehead (bridge) patterns of secondary spread starting from Group 2 (Grp2) or Group 3 (Grp3). Each model here included an independent colonization of Group 1 and Group 5 (indGrp1_5). These estimates were calculated using a neural network approach with a tolerance of 0.05. Weighted 2.5 and 97.5 percentiles are in parentheses.

| Model | Grp2_bridge_indGrp1_5 | Grp2_step_indGrp1_5 | Grp3_bridge_indGrp1_5 | Grp3_step_indGrp1_5 |
| --- | --- | --- | --- | --- |
| Group 1 Ne | 106.77 (11.14, 1595.28) | 97.46 (10.98, 1654.73) | 114.00 (11.37, 1614.37) | 100.43 (11.19, 1557.29) |
| Group 2_Hotel Ne | 228.46 (17.80, 1716.62) | 291.00 (21.85, 1779.14) | 339.42 (19.74, 1755.89) | 351.09 (20.11, 1816.17) |
| Group 2_WestGC Ne | 87.29 (11.25, 1501.65) | 101.95 (11.87, 1616.64) | 119.76 (11.38, 1691.16) | 110.40 (11.82, 1642.14) |
| Group 3_NE Ne | 376.52 (17.63, 1865.90) | 372.10 (17.36, 1845.42) | 358.39 (18.41, 1837.95) | 389.95 (19.61, 1872.32) |
| Group 3_SW Ne | 345.38 (18.01, 1832.60) | 475.75 (23.33, 1856.97) | 366.47 (22.46, 1798.13) | 325.30 (22.78, 1775.46) |
| Group 4 Ne | 118.40 (11.35, 1692.41) | 95.53 (11.14, 1606.02) | 104.48 (11.17, 1632.37) | 130.51 (11.72, 1679.59) |
| Group 5 Ne | 142.74 (12.03, 1767.38) | 102.89 (11.38, 1656.36) | 105.16 (11.24, 1684.72) | 93.36 (11.35, 1587.12) |
| Group 2 migration | 0.001 (0.000, 0.009) | 0.001 (0.000, 0.009) | 0.001 (0.000, 0.009) | 0.001 (0.000, 0.009) |
| Group 3 migration | 0.001 (0.000, 0.009) | 0.001 (0.000, 0.009) | 0.001 (0.000, 0.009) | 0.001 (0.000, 0.009) |
| Bottleneck severity | 0.043 (0.002, 0.230) | 0.047 (0.002, 0.233) | 0.038 (0.002, 0.228) | 0.036 (0.002, 0.227) |

Ne=Effective population size


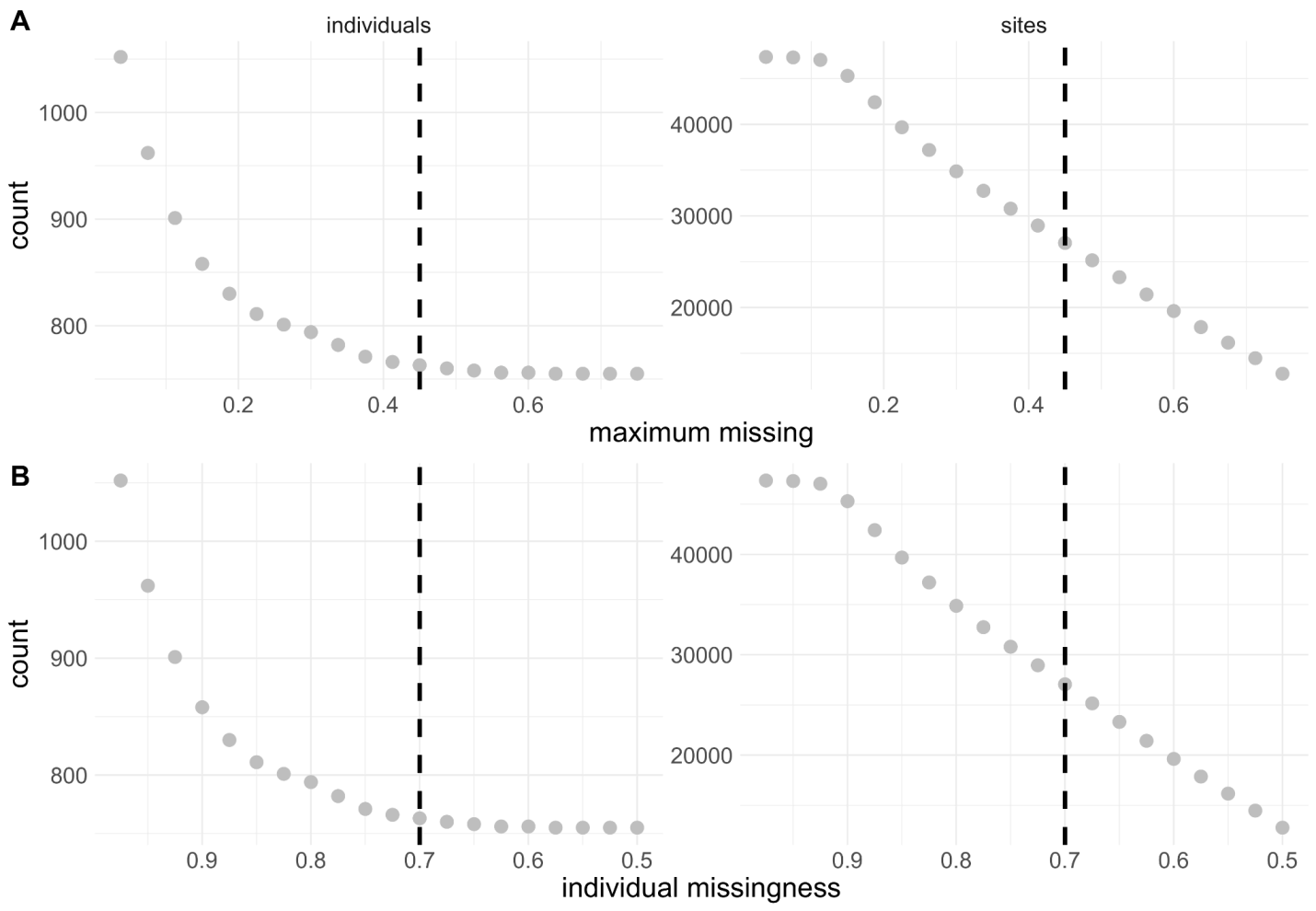


Figure S1. Results from the iterative filtering analysis on the population genetics dataset showing the amount of individuals and loci after each round of filtering with increasing stringency in the amount of A) missingness per loci and B) per individual. The vertical dashed lines indicate the parameters that were used to filter the data.


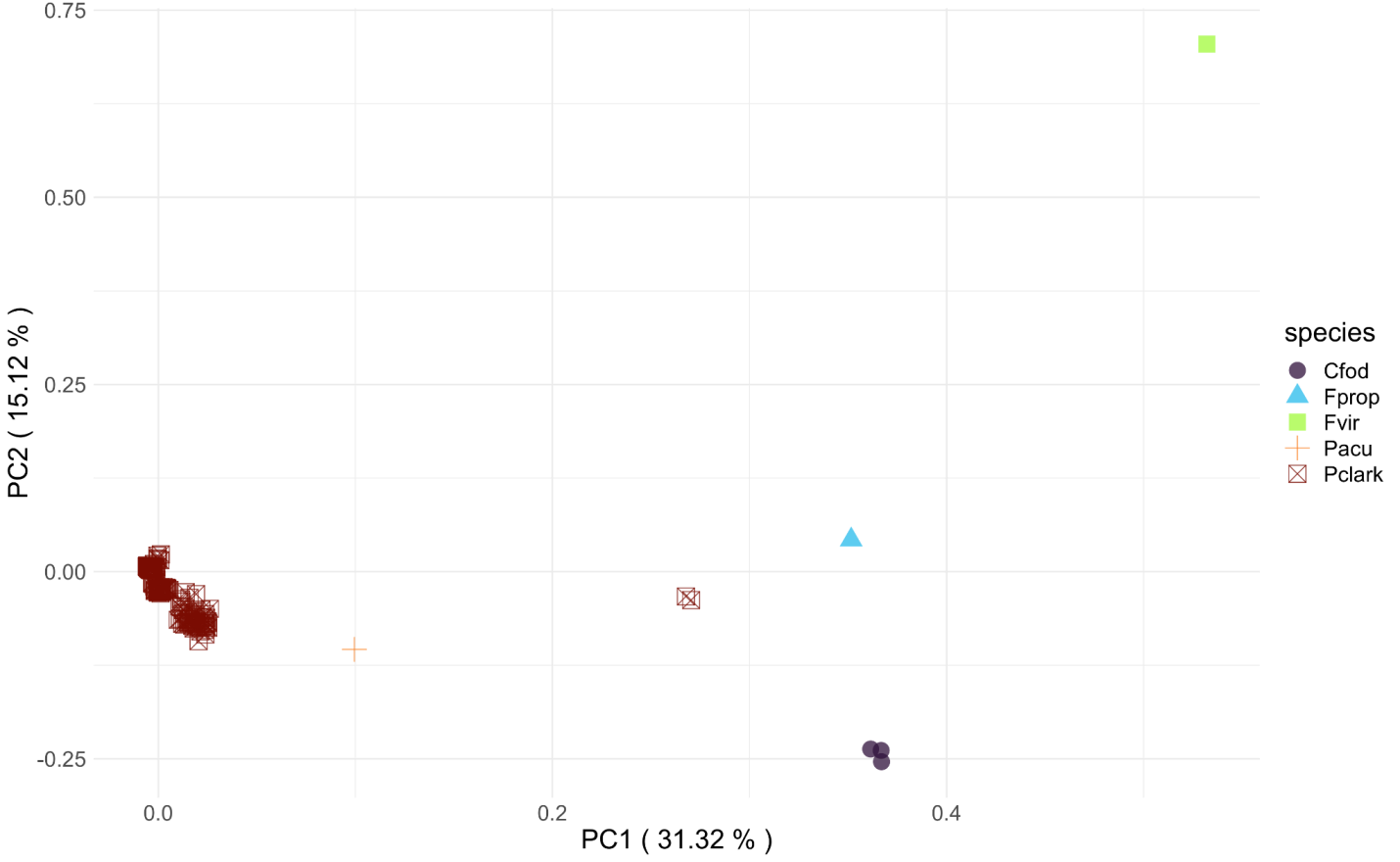


Figure S2. Principal component analysis of multiple crayfish species showing two presumed *P. clarkii* samples that are genetically intermediate based on ordination between other crayfish species, and thus were excluded from analyses. Crayfish species are as follows: *Creaserinus fodiens* (Cfod), *Faxonius propinquus* (Fprop), *Faxonius virilis* (Fvir), *Procambarus acutus* (Pacu), *Procambarus clarkii* (Pclark).

| 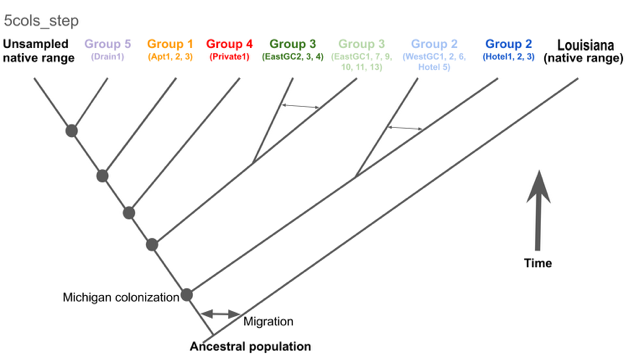 | 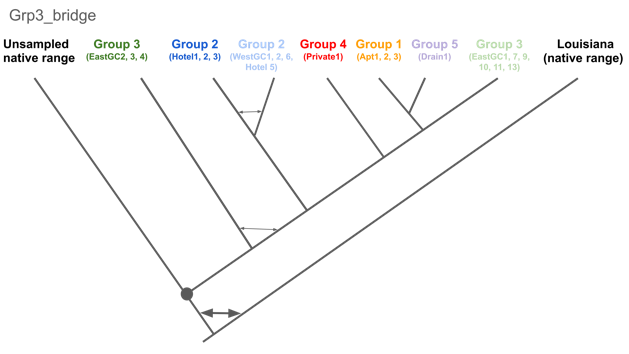 | 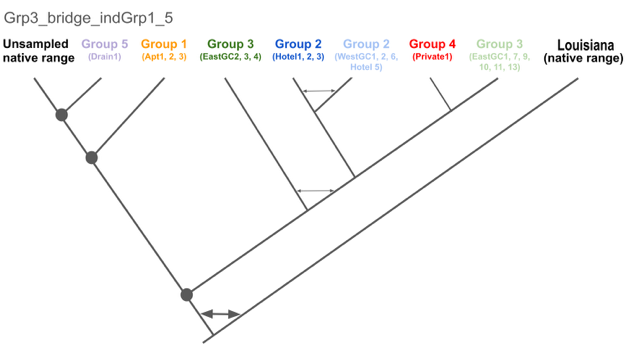 |
| --- | --- | --- |
| 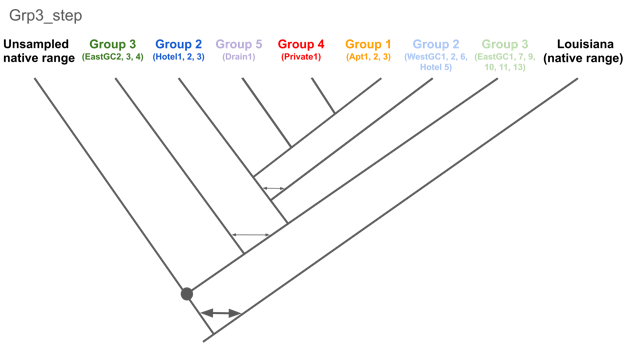 | 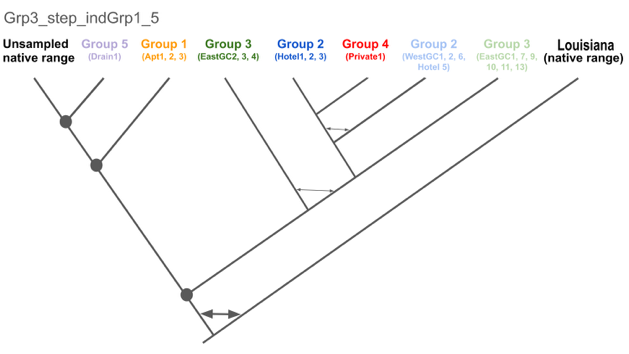 | 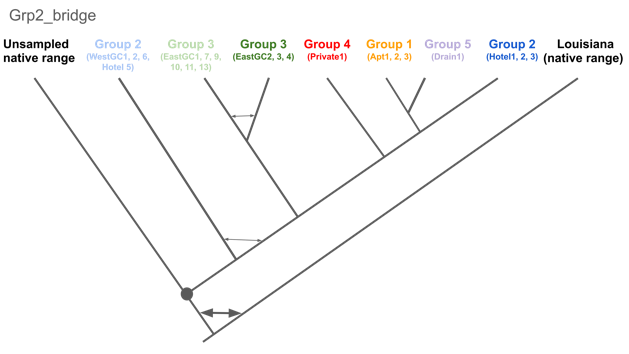 |
| 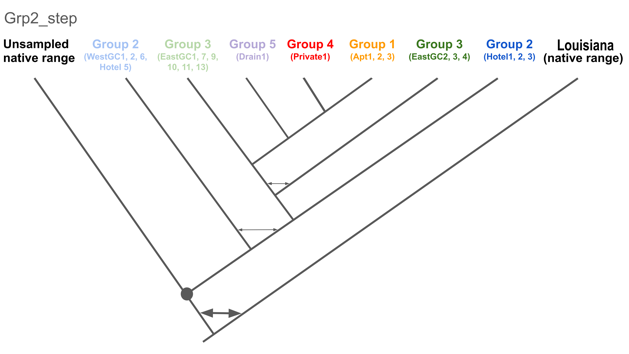 | 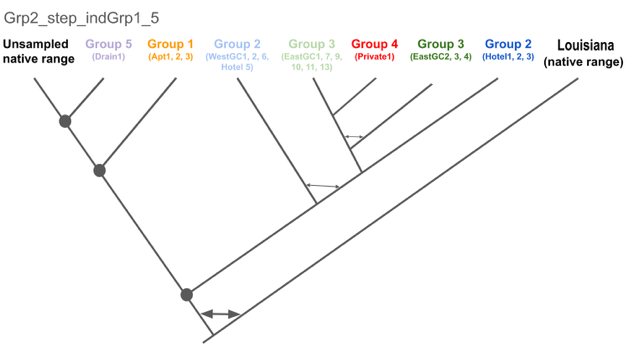 | 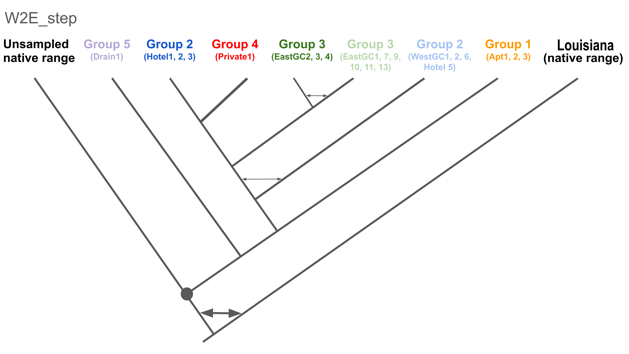 |

Figure S3. Hypothesis trees for demographic modeling. Colonization events in southeastern Michigan are shown in dark circles. Migration between groups is indicated with arrows, the width of the arrow indicative of the relative strength of migration.


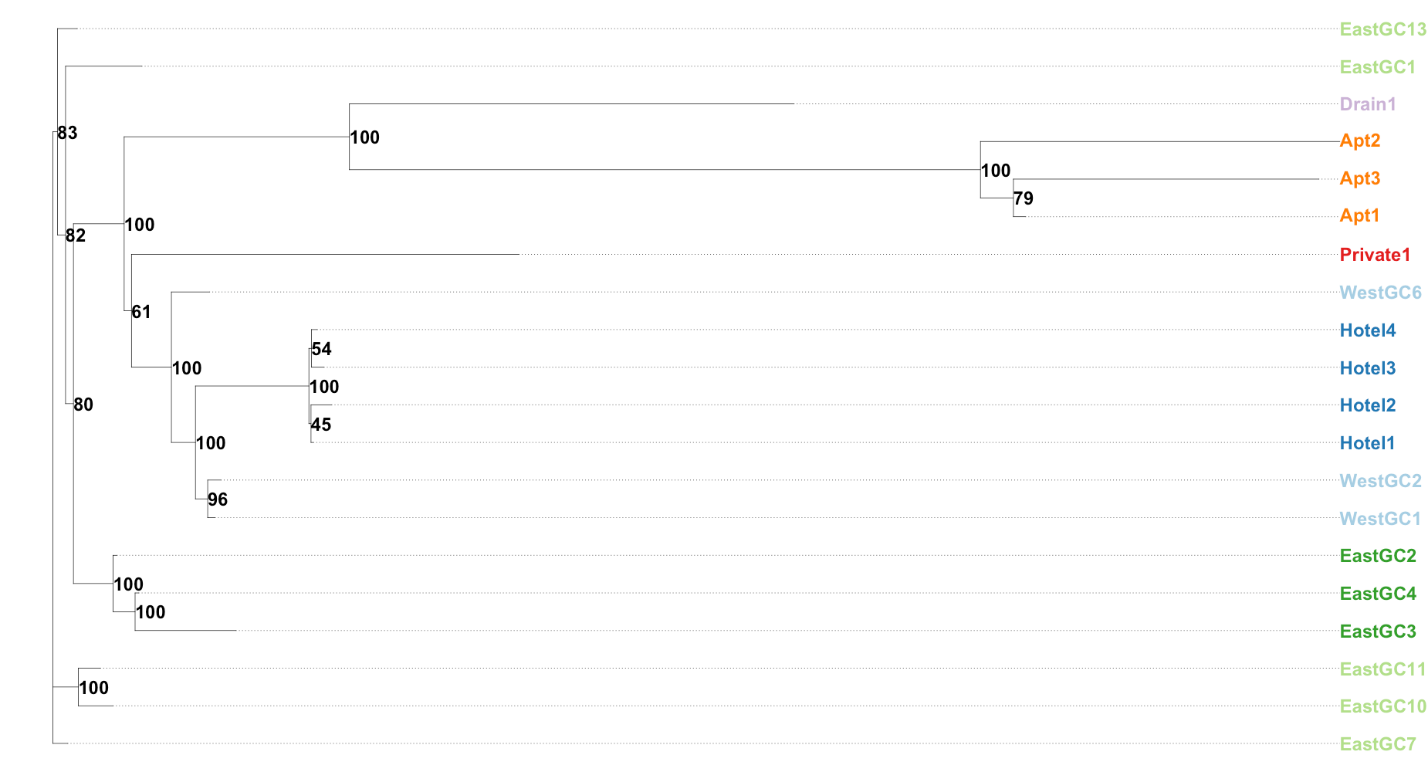


Figure S4. Unrooted neighbor joining tree with node support (percentages) based on 1000 bootstrap replicates. Waterbody abbreviations (see Table S1 for full names) are colored by geographic-genetic Group, as described previously (Fig. 1). Analyses are based on 2,675 SNPs and 757 individuals.


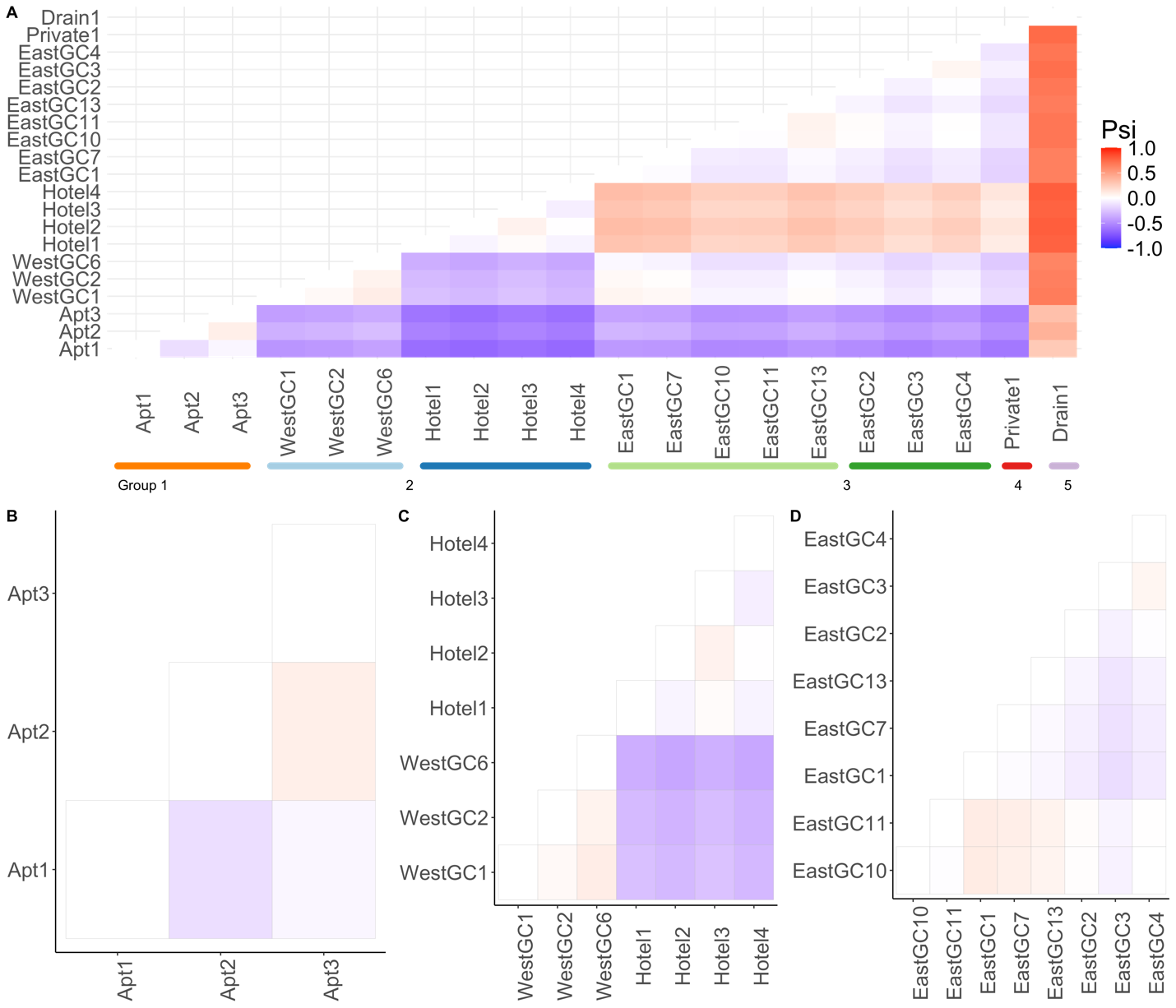


Figure S5. Heat map of pairwise directionality index (Ѱ) values between waterbodies for A) all groups B) Group 1, C) Group 2, and D) Group 3. Groups are indicated with colored bars underneath the waterbody labels in panel A.


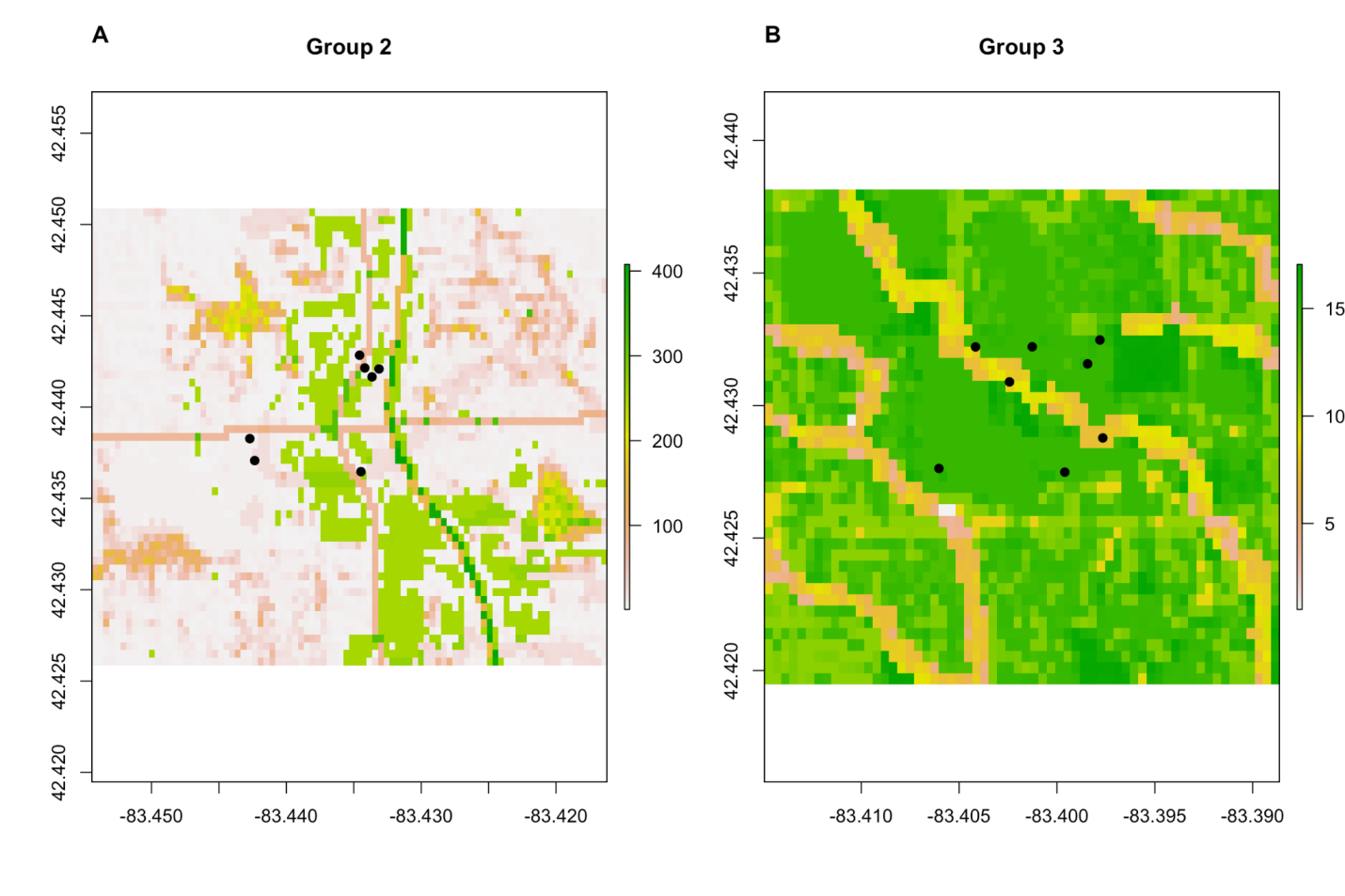


Figure S6. Optimization surfaces results from genetics-based resistance modeling using all environmental features for A) Group 2 and B) Group 3. The color scale indicates resistance and the sampled waterbodies are shown with black circles. Group 2 percent contributions for each environmental layer to the final composite resistance surface are 44.2% NLCD, 34.4% canopy cover, 10.9% hydrography, and 10.5% roads. Group 3 percent contributions for each environmental layer to the final composite resistance surface are 46.5% hydrography, 44.1% NLCD, 5.7% canopy, and 3.7% roads.


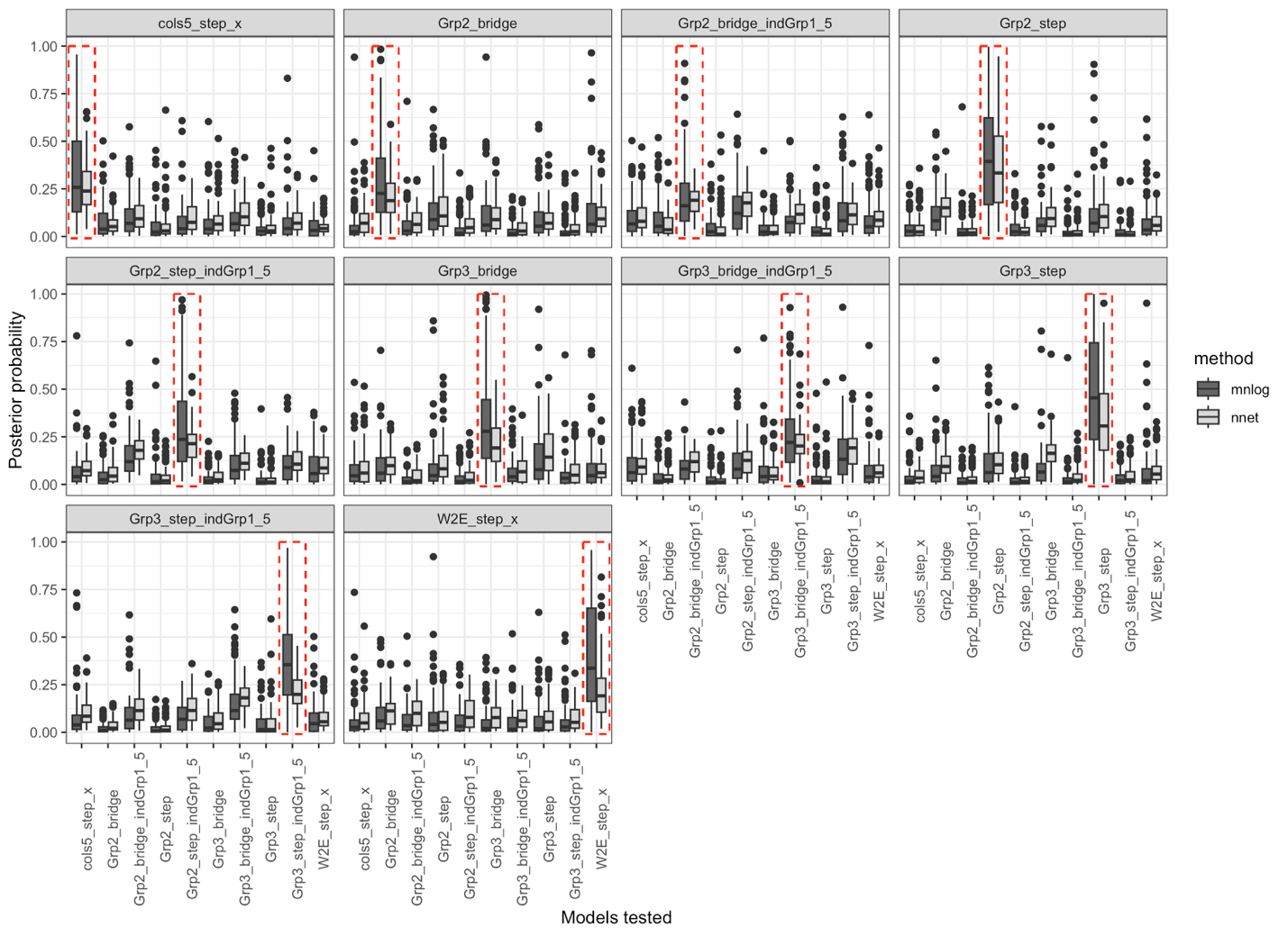


Figure S7. Boxplots of posterior probabilities from leave-one-out cross-validation replicates using multinomial logistic regression (mnlog, dark grey) and neural network (nnet, light grey) methods. The title of each plot is the true (simulated) model, the x-axis shows all the models tested, and red dashed rectangles are around the simulated model. For model details see Fig. S3.

A

B

Figure S8. Posterior density plots of parameter estimates for the best supported models originating from A) Group 2 and B) Group 3, all with independent Group 1 and 5 introductions. Parameter estimates are based on a tolerance of 0.05 (5000 accepted replicates). Stepping-stone models are in darker hued lines, while bridgehead models are in lighter hued lines. Estimates based on a neural network method are indicated by wide lines and localized linear based estimates are marked by narrower lines. The priors are shown as a dotted black line. Ten focal parameters are shown here including estimated contemporary effective population sizes (Ne) for the five groups. Groups 2 and 3 are split into two additional groups based on our PCA and DAPC results: Group 2 into the Hotel locations and West Golf Course locations (WestGC), Group 3 into the locations to the southwest (SW) and northeast (NE).

A

B

C

D

Figure S9. Parameter cross-validation plots for A) Group 2 bridgehead B) Group 2 stepping-stone C) Group 3 bridgehead D) Group 3 stepping-stone models each with independent Group 1 and 5 introductions. Ten focal parameters are shown here including estimated contemporary effective population sizes (Ne) for the five groups. Groups 2 and 3 are split into two additional groups based on our PCA and DAPC results: Group 2 into the Hotel locations and West Golf Course locations (WestGC), Group 3 into the locations to the southwest (SW) and northeast (NE). The dashed red line indicates a 1:1 relationship that would be indicative of a high correlation between simulated (x-axis) and estimated (y-axis) parameter values.
